# Author Correction: Enhanced oxygen reduction with single-atomic-site iron catalysts for a zinc-air battery and hydrogen-air fuel cell

**DOI:** 10.1038/s41467-022-28495-2

**Published:** 2022-02-11

**Authors:** Yuanjun Chen, Shufang Ji, Shu Zhao, Wenxing Chen, Juncai Dong, Weng-Chon Cheong, Rongan Shen, Xiaodong Wen, Lirong Zheng, Alexandre I. Rykov, Shichang Cai, Haolin Tang, Zhongbin Zhuang, Chen Chen, Qing Peng, Dingsheng Wang, Yadong Li

**Affiliations:** 1 Department of Chemistry, Tsinghua University, 100084 Beijing, China; 2 Beijing Guyue New Materials Research Institute, Beijing University of Technology, 100124 Beijing, China; 3 Beijing Synchrotron Radiation Facility, Institute of High Energy Physics, Chinese Academy of Sciences, 100049 Beijing, China; 4 State Key Laboratory of Coal Conversion, Institute of Coal Chemistry, Chinese Academy of Sciences, 030001 Taiyuan, China; 5 Mössbauer Effect Data Center, Dalian Institute of Chemical Physics, Chinese Academy of Sciences, 116023 Dalian, China; 6 State Key Laboratory of Advanced Technology for Materials Synthesis and Processing, Wuhan University of Technology, 430070 Wuhan, China; 7 State Key Lab of Organic-Inorganic Composites and Beijing Advanced Innovation Center for Soft Matter Science and Engineering, Beijing University of Chemical Technology, 100029 Beijing, China

**Keywords:** Electrocatalysis, Batteries, Fuel cells, Electrocatalysis, Batteries, Fuel cells

Correction to: *Nature Communications* 10.1038/s41467-018-07850-2, published online 21 December 2018.

In this article the wrong figure appeared as Supplementary Figure 47; the figure should have appeared as shown below. 

The original article has been corrected.

## Supplementary information

Updated Supplementary information

