# Supplementary material for: Enhanced oxygen reduction with single-atomic-site iron catalysts for a zinc-air battery and hydrogen-air fuel cell
Source: Nat Commun. 2018 Dec 21;9:5422. doi: 10.1038/s41467-018-07850-2 (PMC6303331; doi:10.1038/s41467-018-07850-2)
Supplement: Supplementary file 1 — Supplementary Information [file 41467_2018_7850_MOESM1_ESM.pdf]

Enhanced oxygen reduction with single-atomic-site iron catalysts for a zinc-air battery and hydrogen-air fuel cell

Supplementary Information

chen et al.

Supplementary Note

Reagents. Zinc nitrate hexahydrate (98%), bis(4-hydroxyphenyl) sulfone (98%), phosphonitrilic chloride trimer (98%), bis(4-aminophenyl) ether (98%), nafion D-521 dispersion (5% w/w in water and 1-propanol), 2-methylimidazole (98%) and commercial Pt/C (20 wt% metal) were obtained from Alfa Aesar. Iron (III) nitrate nonahydrate (98.5%), N,N-diethylethanamine (analytical grade), methanol (analytical grade), N,N-dimethylformamide (analytical grade) and KOH (analytical grade) were purchased from Sinopharm Chemical. The distilled water used in all experiments was obtained through ion-exchange and filtration. All reagents were used without any further purification.

XAFS measurements and data analysis details. The ex stiu XAFS spectra at Fe K-edge was acquired at 1W1B station in Beijing Synchrotron Radiation Facility (BSRF, operated at 2.5 GeV with a maximum current of 250 mA). The data of Fe-SAs/NPS-HC sample was recorded in fluorescence excitation mode using a Lytle detector. Fe foil and Fe_2O_3 were used as references and measured in a transmission mode using ionization chamber. All spectra were collected in ambient conditions. The acquired EXAFS data were processed according to the standard procedures using the ATHENA module implemented in the IFEFFIT software packages. The k^3-weighted EXAFS spectra were obtained by subtracting the post-edge background from the overall absorption and then normalizing with respect to the edge-jump step. Subsequently, k^3-weighted $\chi(k)$ data of Fe K-edge were Fourier transformed to real (R) space using a Hanning window function (dk=1.0 $Å^{-1}$) to separate the EXAFS contributions from different coordination shells. To obtain the quantitative structural parameters around central atoms, least-squares curve parameter fitting was performed using the ARTEMIS module of IFEFFIT software packages. In situ XAFS measurements were used to obtain the change of valence state for Fe element in the catalyst during reaction. The data of Fe-SAs/NPS-HC sample were recorded in fluorescence excitation mode using a solid-state detector. Electrochemical measurements were conducted on a computer-controlled electrochemical analyzer. The Fe-SAs/NPS-HC catalyst modified carbon paper was used as working electrode, graphite rod as counter electrode and Ag/AgCl (saturated KCl solution) as reference electrode. A home-made electrochemical cell was used for in-situ XAFS measurements.

Electrochemical measurements for ORR.

The electron transfer number (n) was determined by the Koutecky-Levich equation:

$$\frac{1}{J} = \frac{1}{J_L} + \frac{1}{J_K} = \frac{1}{B\omega^{\frac{1}{2}}} + \frac{1}{J_K}$$

$$\mathrm{B} = 0.62\mathrm{n}FC_0D_0^{\frac{2}{3}}V^{-\frac{1}{6}}$$

where J is the measured current density, J_K and J_L are the kinetic and limiting current densities, ω is the angular velocity

of the disk, n is the overall number of electrons transferred in oxygen reduction, F is the Faraday constant (96485 C mol^{-1}), C_0 is the bulk concentration of O_2 (1.2×10^{-6} mol cm^{-3}), D_0 is the diffusion coefficient of O_2 (1.9×10^{-5} cm^2 s^{-1}), and V is the kinematic viscosity of the electrolyte (0.01 cm^2 s^{-1}), and k is the electron transfer rate constant.

The hydrogen peroxide yield (H_2O_2 %) and the electron transfer number (n) were calculated with the following equations:

$$H_2O_2(\%) = 200 \times \frac{\frac{I_R}{N}}{I_D + \frac{I_R}{N}}$$

$$n = 4 \times \frac{I_D}{\frac{I_R}{N} + I_D}$$

where I_D is the disk current, I_R is the ring current, and N is the ring collection efficiency and N=0.37.

The mass activities (MAs) and turnover frequencies (TOFs) of the catalysts were calculated by normalizing them with total metal content according to the following equations:

$$\text{MA [A/g]} = \frac{j_k}{m_0 \times w_{metal}}$$

$$\text{TOF [e/(site•s)]} = \frac{j_k}{n_{metal} \times F} = \frac{j_k \times M_{metal}}{m_{metal} \times F} = \frac{j_k \times M_{metal}}{m_0 \times w_{metal} \times F}$$

Where j_k is the kinetic current, m_0 is the mass loading of catalysts on glassy carbon electrode surface, w_{metal} is the mass concentration of metal in the catalysts, n_{metal} is the mol amount of metal in the catalysts, m_{metal} is the mass of metal in the catalysts, M_{metal} is the atomic weight of metal, and F is the Faraday constant (96485 C mol^{-1}).

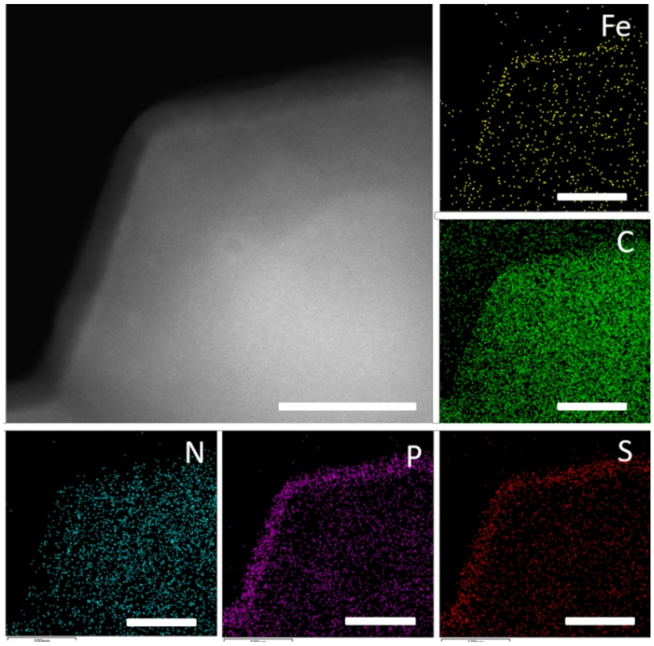

Supplementary Figure 1 | HAADF-STEM image and corresponding EDS element mapping analysis of ZIF-8/Fe@PZS. The EDS element mapping images display that the distribution of S (red), P (purple) and Fe (yellow) are evenly dispersed in coating layer of ZIF-8/Fe@PZS. And C (green) and N (cyan) are uniformly distributed in the entire ZIF-8/Fe@PZS. Scale bar, 100 nm.

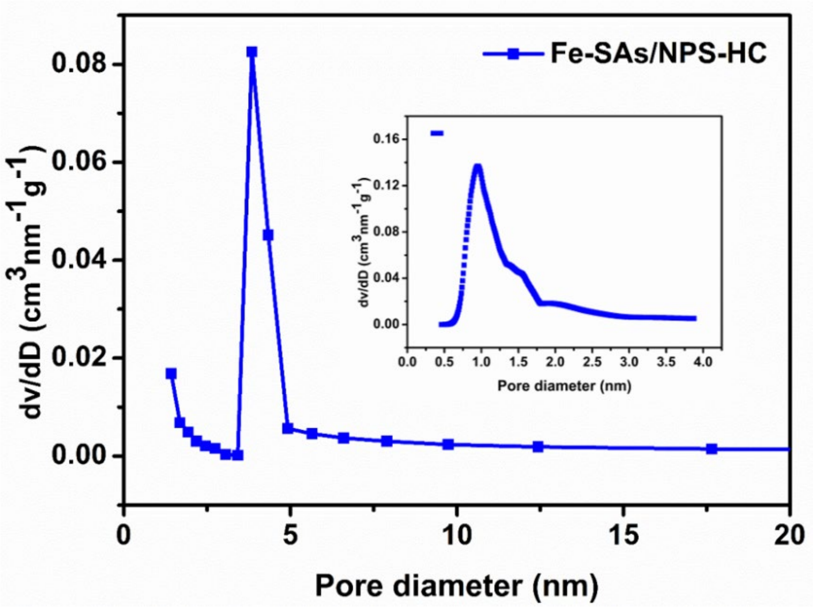

Supplementary Figure 2 | The pore-size distributions of Fe-SAs/NPS-HC. From the corresponding pore-size distribution calculated by the Barrett–Joyner–Halenda (BJH) method, a main peak at ~4.5 nm indicates a mesoporous structure. Inset: a peak around 1.1 nm of pore-size distribution calculated by the Horvath–Kawazoe method is detected, indicating a microcellular structure.

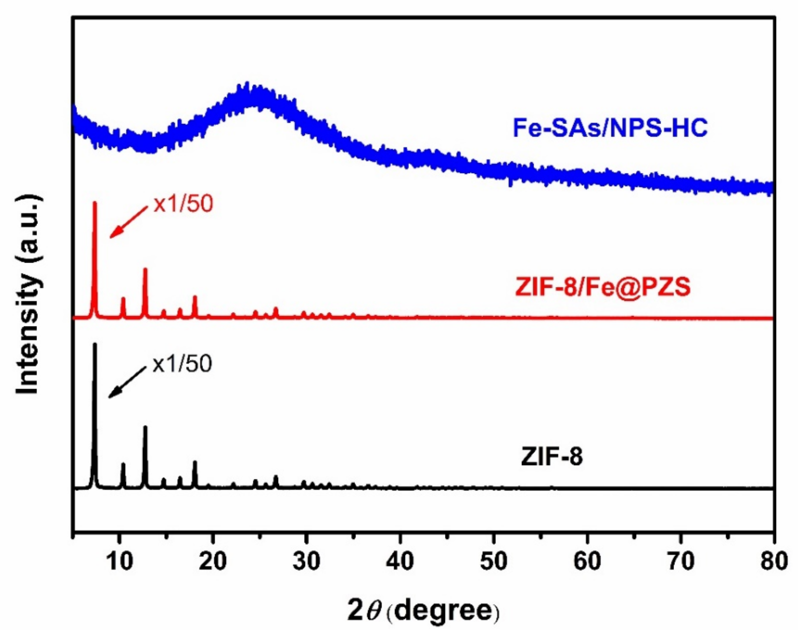

Supplementary Figure 3 | XRD patterns of Fe-SAs/NPS-HC, ZIF-8/Fe@PZS and ZIF-8. The XRD pattern of Fe-SAs/NPS-HC (blue line) demonstrates no signals for metallic Fe species are detected. The XRD pattern of ZIF-8/Fe@PZS (red line) is similar to pure ZIF-8 (black line).

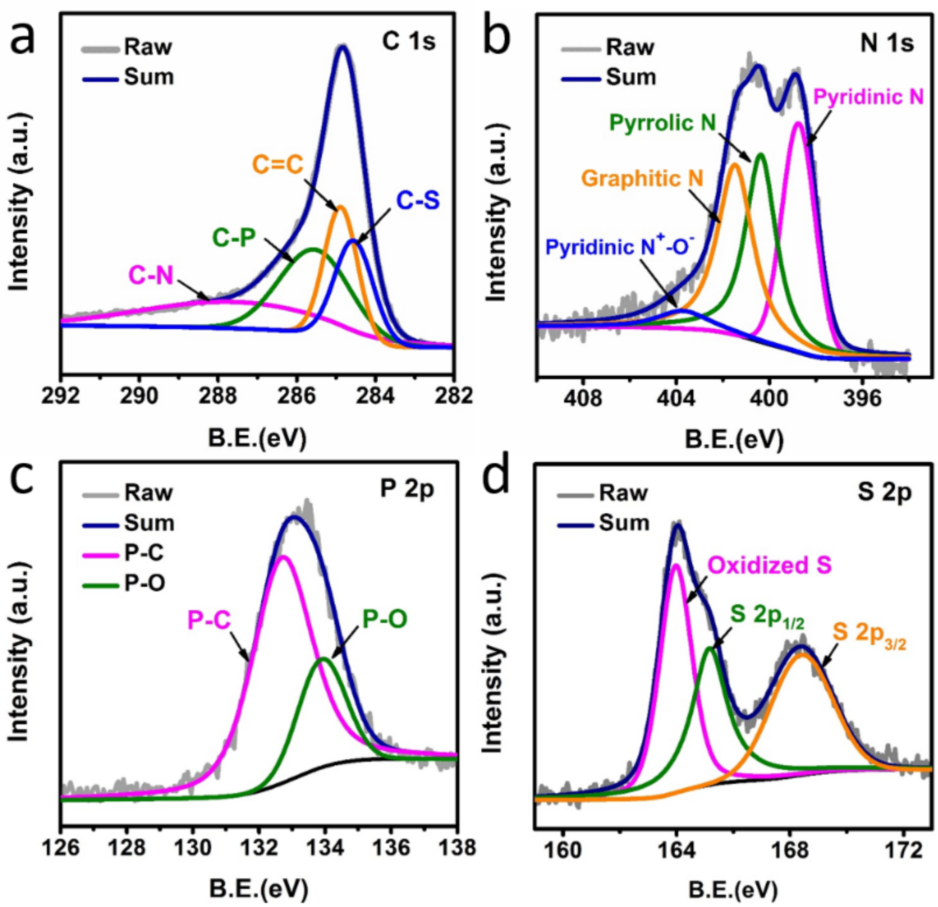

Supplementary Figure 4 | The X-ray photoelectron spectroscopy (XPS) spectra of Fe-SAs/NPS-HC. a The C 1*s* spectrum; **b** N 1*s* spectrum; **c** P 2*p* spectrum; **d** S 2*p* spectrum.

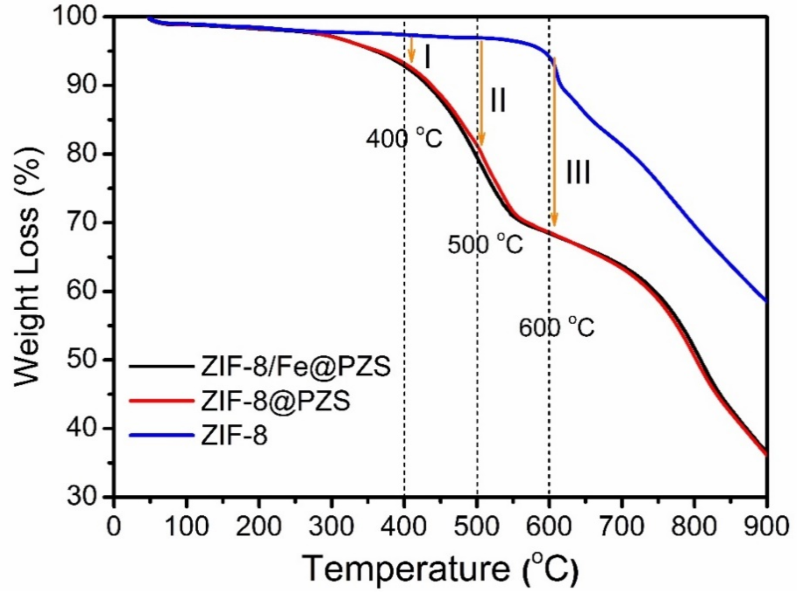

Supplementary Figure 5 | Thermogravimetric (TGA) analysis of ZIF-8/Fe@PZS, ZIF-8@PZS and pure ZIF-8.

Compared with pure ZIF-8, TGA analysis of ZIF-8/Fe@PZS (Fe-SAs/NPS-HC catalyst precursor) and ZIF-8@PZS (NPS-HC catalyst precursor) show that obvious gasification weight loss platforms at 400, 500 and 600 ºC, respectively.

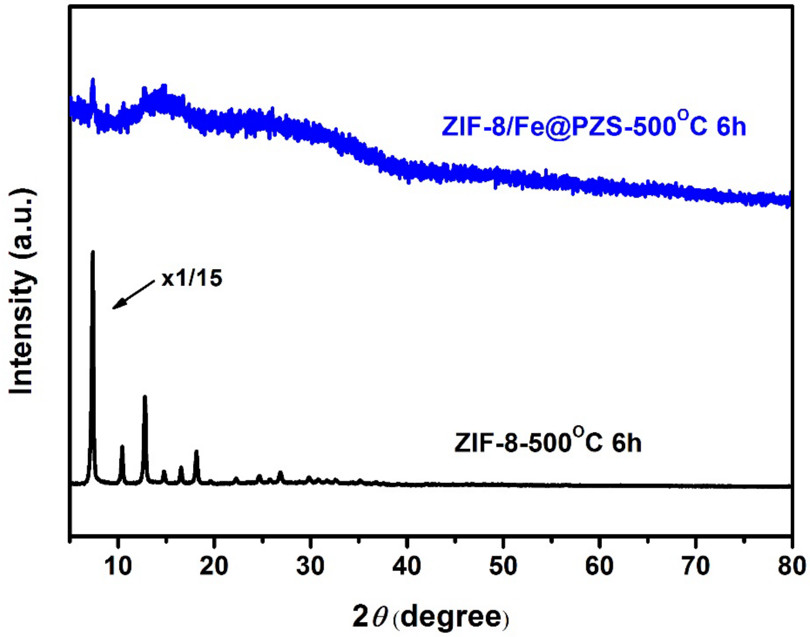

Supplementary Figure 6 | XRD patterns of ZIF-8/Fe@PZS and ZIF-8 with thermal treatment at 500 ºC for 6 h.

Comparison for ZIF-8 with thermal treatment at 500 ºC for 6 h, the XRD pattern of ZIF-8/Fe@PZS under identical conditions shows that characteristic peak of ZIF-8 significantly decreases.

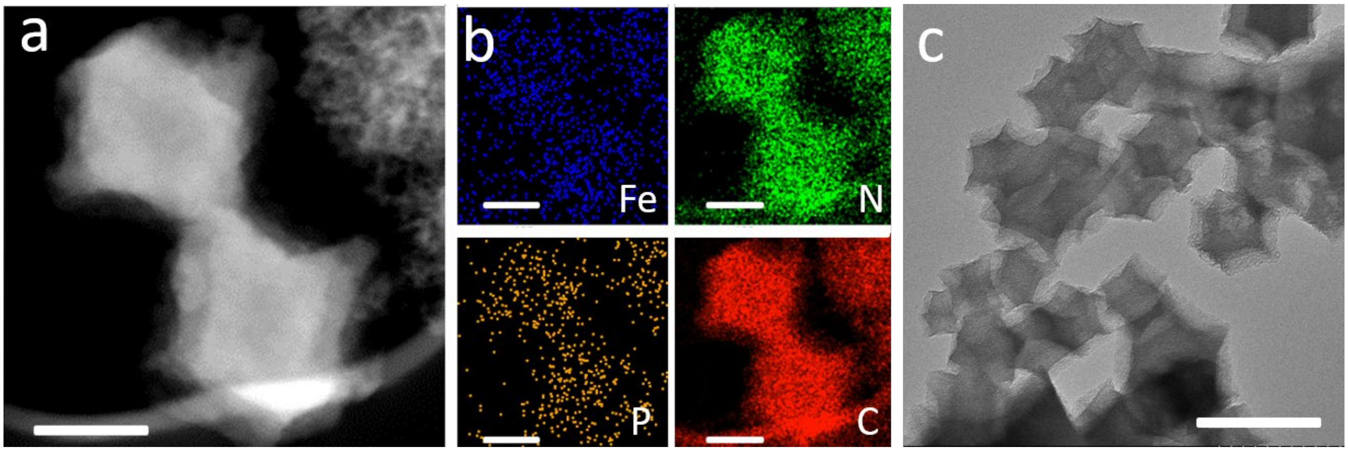

Supplementary Figure 7 | Characterization of Fe-SAs/NP-C and N-C. a HAADF-STEM image of Fe-SAs/NPS-C shows a solid morphology. Scale bar, 100 nm. **b** The corresponding EDS element maps display the uniform distribution of Fe (blue), N (green), P (yellow) and C (red) of Fe-SAs/NP-C. Scale bar, 100 nm. **c** TEM image of N-C shows the dodecahedral structure. Scale bar, 200 nm.

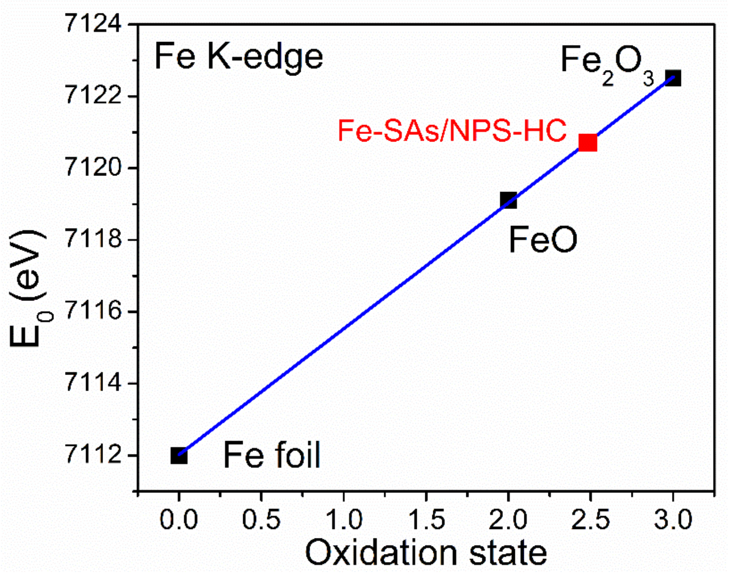

Supplementary Figure 8 | Average oxidation state of Fe in the Fe-SAs/NPS-HC. The oxidation state of Fe could be reflected through the absorption edge of Fe K-edge. The average state of the Fe-SAs/NPS-HC was ~2.5, in comparison with Fe foil (0), FeO and Fe_2O_3.

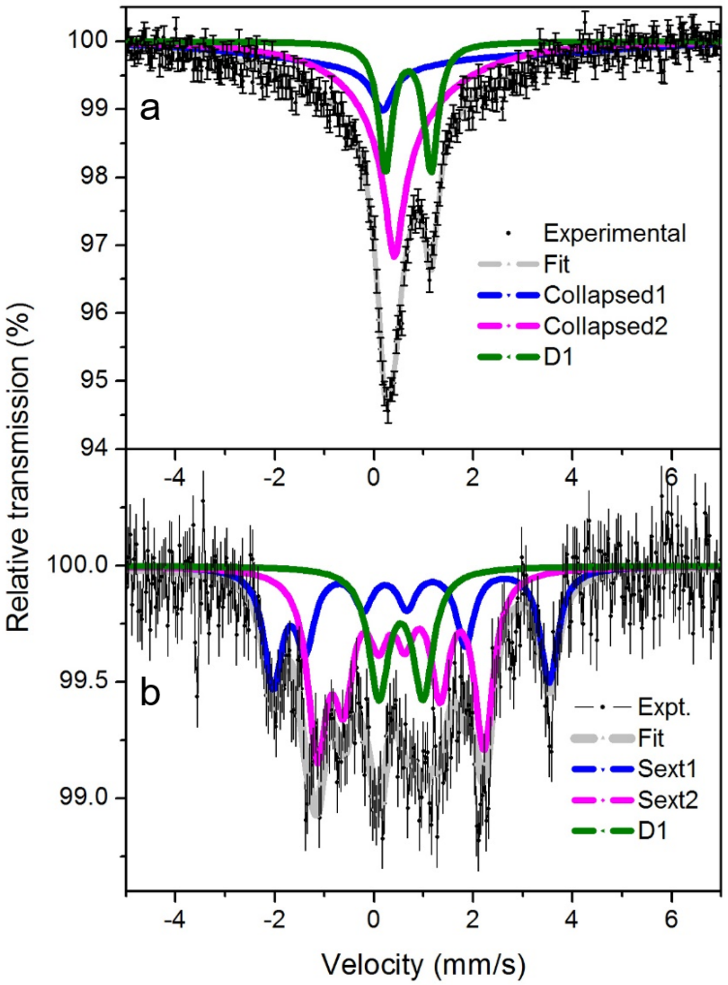

Supplementary Figure 9 | Mössbauer spectroscopy of Fe-SAs/NPS-HC. a The room-temperature (RT) spectrum exhibits the slowly decaying wings indicating the relaxational nature of two magnetically collapsed subspectra complemented with a minor paramagnetic doublet D1. **b** The spectrum measured at 78 K consist of the same minor doublet and two magnetically split subspectra with mixed (M+Q) interaction, both showing the large quadrupole lineshifts identifying the orientation of the hyperfine fields normal to the plane of Fe-N_4, i.e. H ‖ Vzz with positive values of Vzz for both Fe(II) and Fe(III) sites (Vzz ⊥ Fe(II)-N_4 and Vzz ⊥ Fe(III)-N_4).

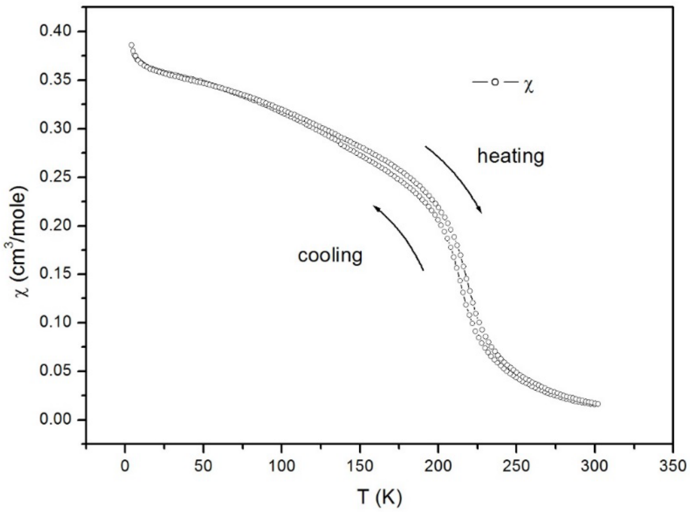

Supplementary Figure 10 | Temperature dependence of magnetic susceptibility in the applied field of 1 kOe for the Fe-SAs/NPS-HC, showing the ferromagnetic ordering in the system of S=1 and S=1/2 species below T_C. The Curie temperature of 215 K was determined by fitting a sigmoidal function to the $\chi(T)$ curves.

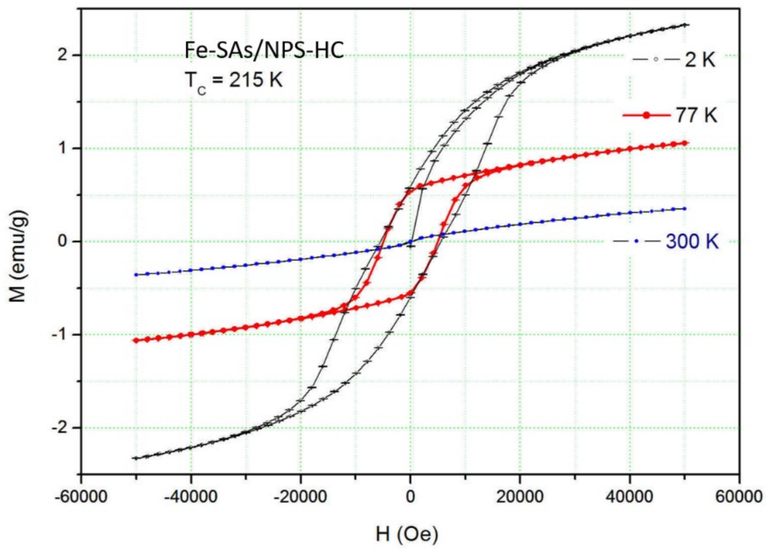

Supplementary Figure 11 | M-H loops measured below (2 K and 77 K) and above (at 300 K) of the Curie point of 215K for the Fe-SAs/NPS-HC. The magnetic hysteresis at 77 K is characterized by the saturation remanence of 0.5 emu g^{-1} and of coercive force of 5000 Oe.

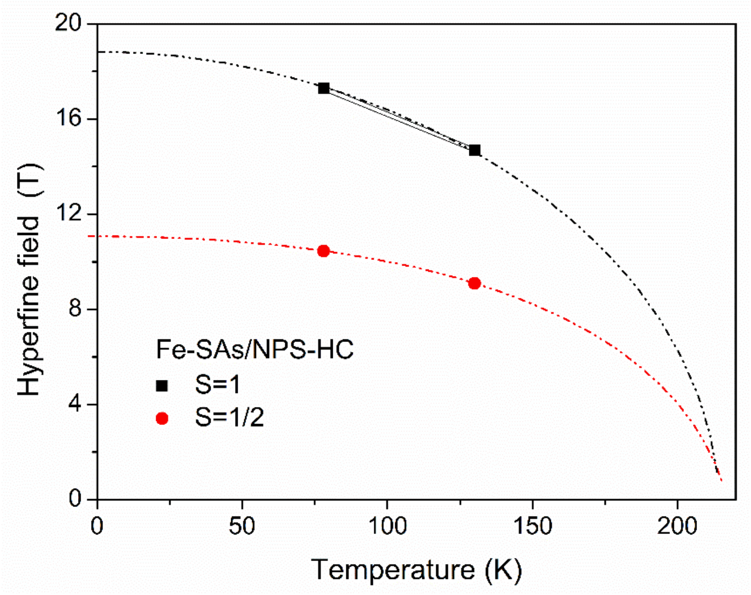

Supplementary Figure 12 | Hyperfine fields in the Fe-SAs/NPS-HC at 77 K and at 130 K. The dotted lines are the eye guides to show the saturation fields (at 0 K).

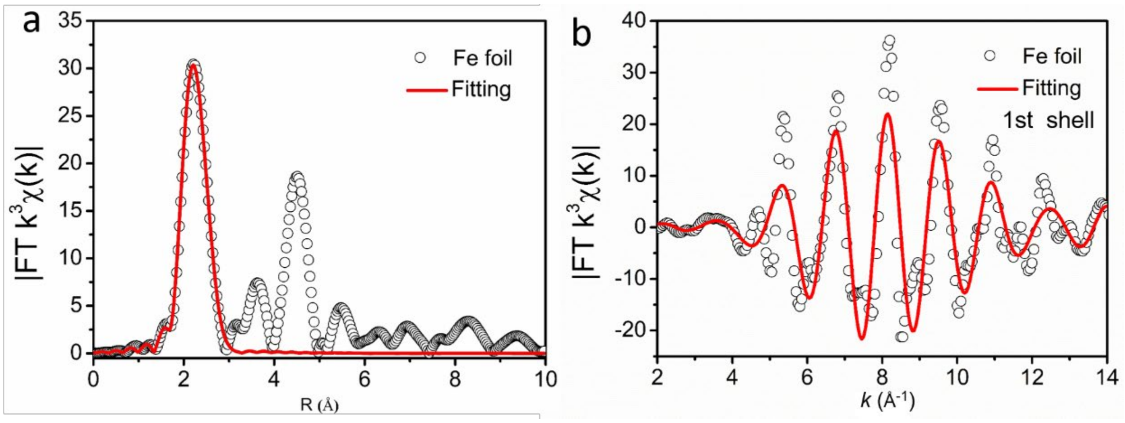

Supplementary Figure 13 | EXAFS fitting curves of Fe foil. a EXAFS *R* space fitting curves of the first shell Fe-Fe shell and **b** EXAFS *k* space signal of the first Fe-Fe shell of Fe foil.

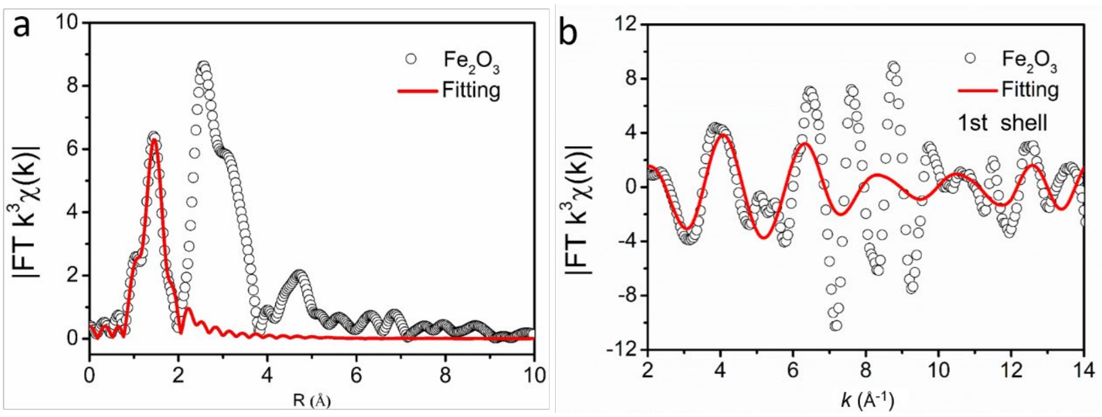

Supplementary Figure 14 | EXAFS fitting curves of Fe_2O_3. a EXAFS *R* space fitting curves for the Fe-O shell and **b** EXAFS *k* space signal from the first Fe-O shell of Fe_2O_3

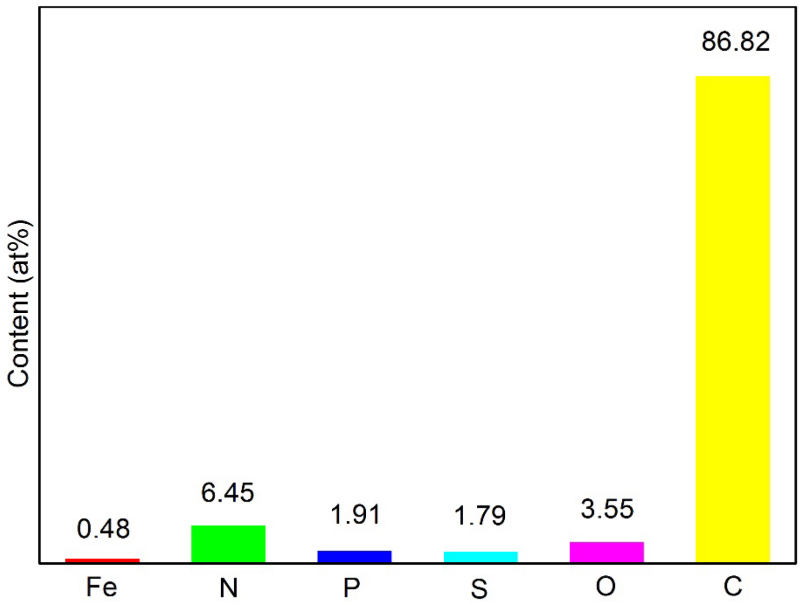

Supplementary Figure 15 | The atomic content percentages of Fe, N, P, S, O and C in Fe-SAs/NPS-HC measured by XPS analysis.

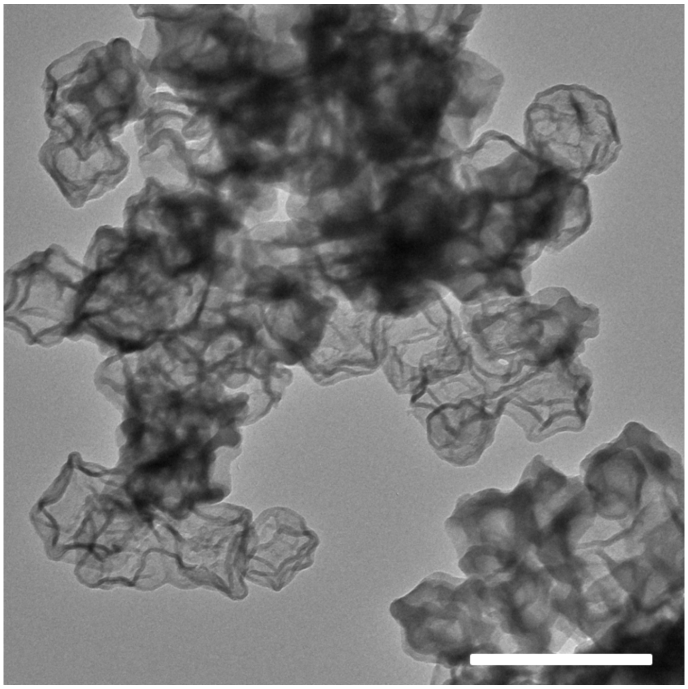

Supplementary Figure 16 | The TEM image of NPS-HC. TEM image shows that NCS-HC displays the hollow shell structure with a dodecahedral morphology Scale bar, 500 nm.

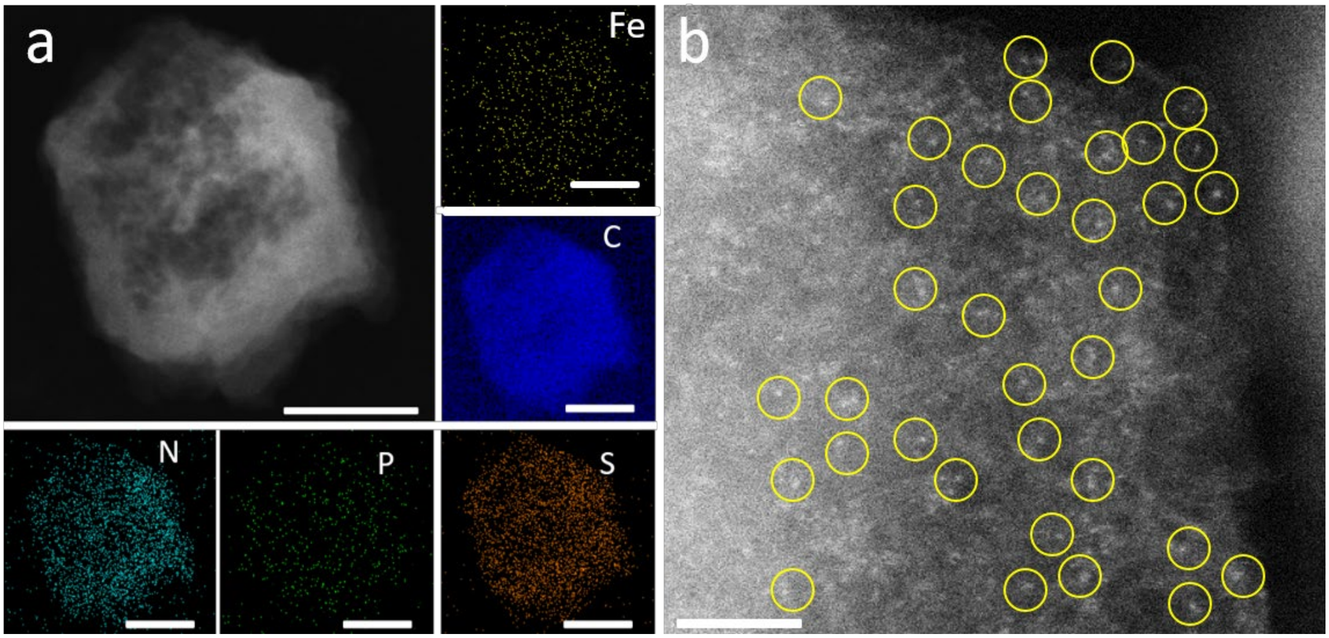

Supplementary Figure 17 | Characterization of Fe-SAs/NPS-C. a HAADF-STEM image of Fe-SAs/NPS-C shows a solid rhombododecahedron morphology, and the corresponding EDS element maps display the uniform distribution of Fe (yellow), N(cyan), S(orange), P(green) and C(blue). Scale bar, 100 nm. **b** AC HAADF-STEM image shows that isolated Fe single atoms are observed and highlighted by yellow circles. Scale bar, 2 nm.

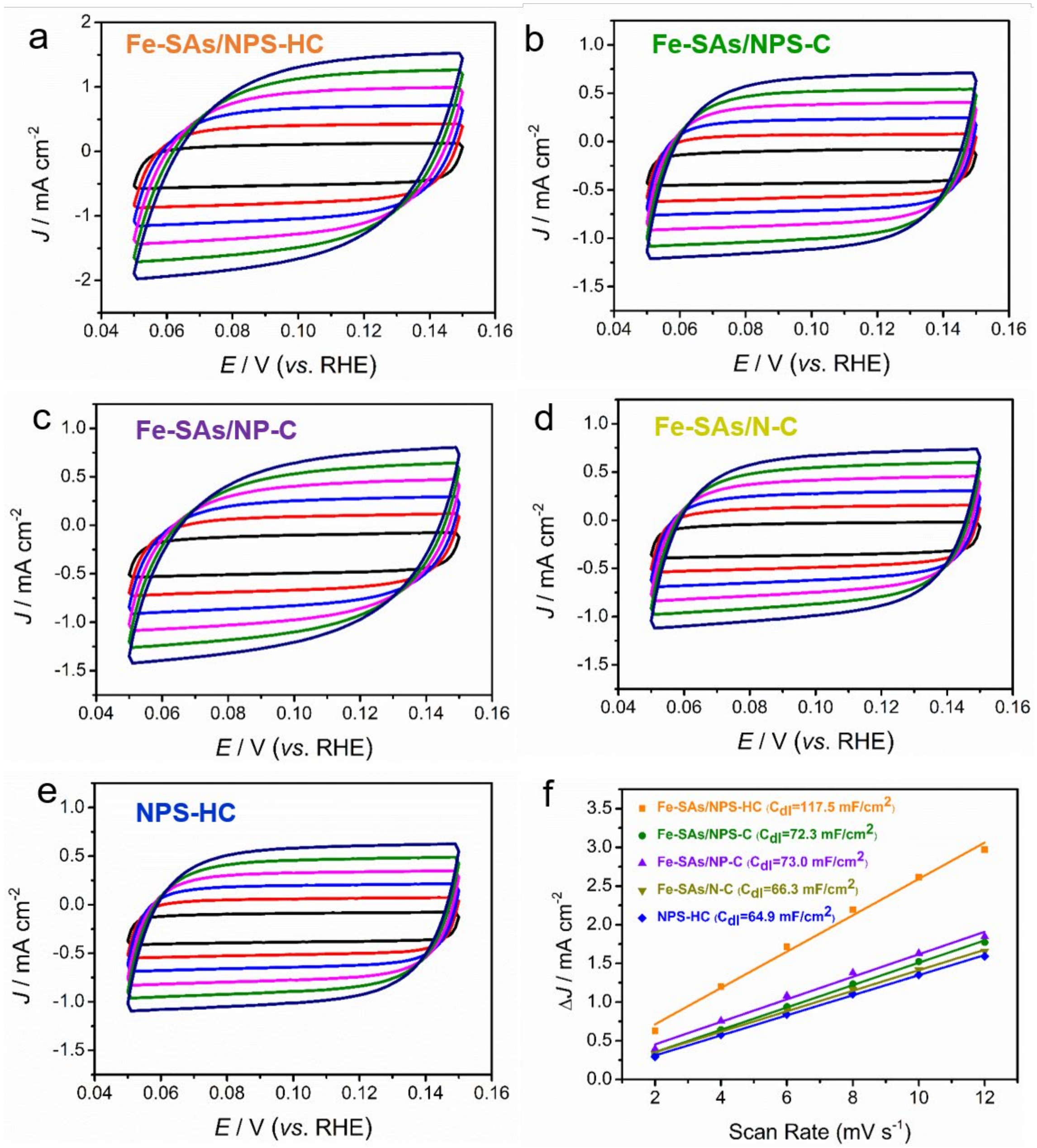

Supplementary Figure 18 | Cyclic voltammograms (CVs) for ORR at different scan rates in the range of no Faradaic processes for measuring C_{dl}. a-e CV curves of the Fe-SAs/NPS-HC, Fe-SAs/NPS-C, Fe-SAs/NP-C, Fe-SAs/N-C and NPS-HC in 0.1 M KOH solution in the region of 0.05~0.15 V vs. RHE for ORR. **f** CV fitting curves of Fe-SAs/NPS-HC, Fe-SAs/NPS-C, Fe-SAs/NP-C, Fe-SAs/N-C and NPS-HC catalysts in 0.1 M KOH for ORR.

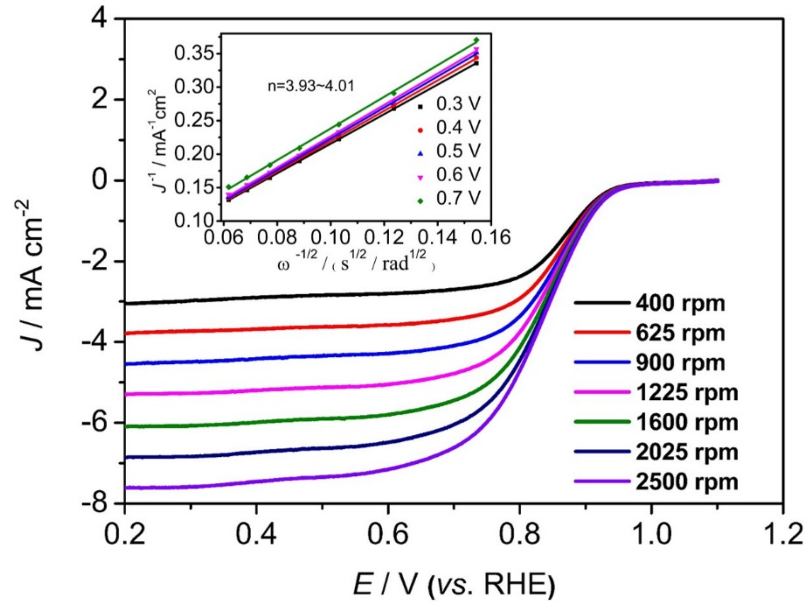

Supplementary Figure 19 | The ORR polarization curves of 20% Pt/C at different rotating rates (inset: K-L plots and electron transfer numbers).

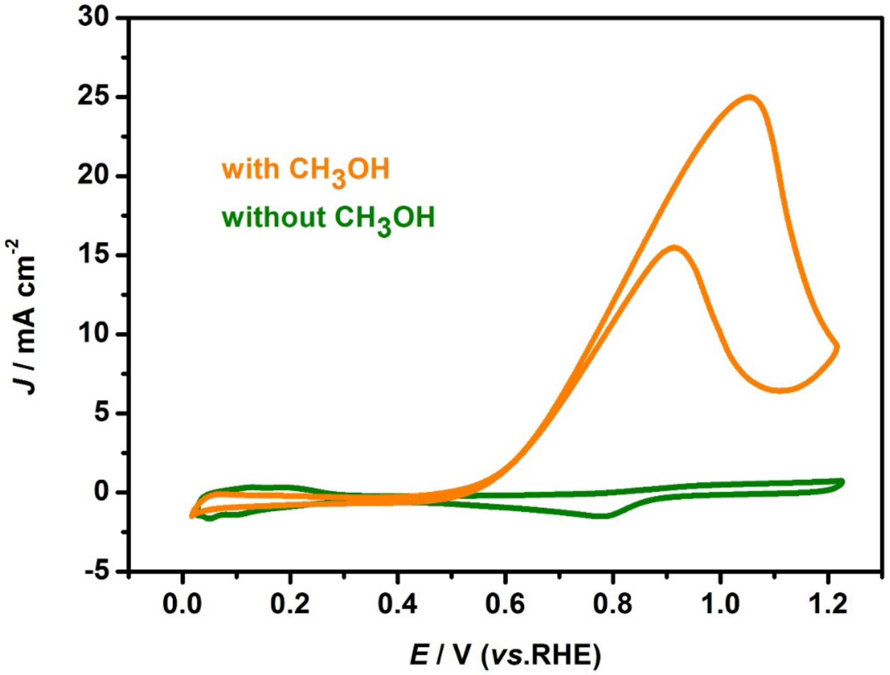

Supplementary Figure 20 | The tolerance to methanol crossover test of the commercial Pt/C. CV curves of the 20 wt% Pt/C are measured in O_2-saturated 0.1 M KOH with and without 1.0 M CH_3OH at a scan rate of 50 mV s^{-1}.

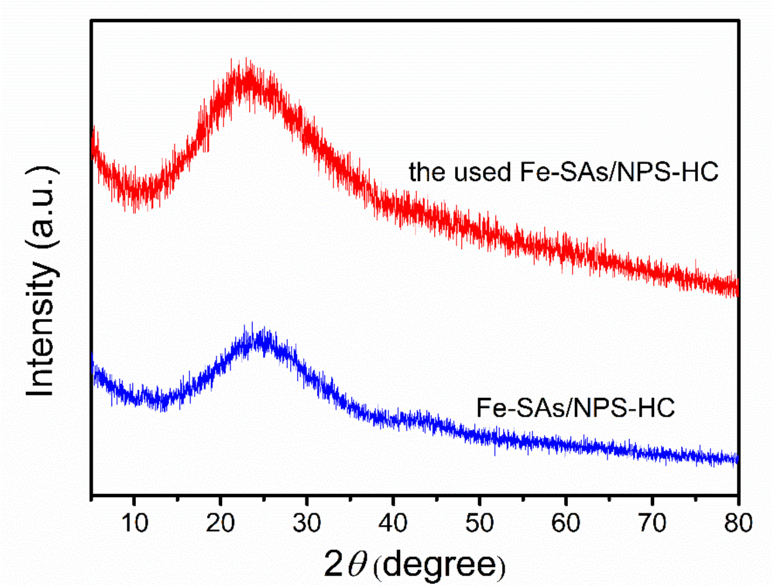

Supplementary Figure 21 | The XRD pattern of the used Fe-SAs/NPS-HC catalyst after durability test compared with the fresh Fe-SAs/NPS-HC catalyst.

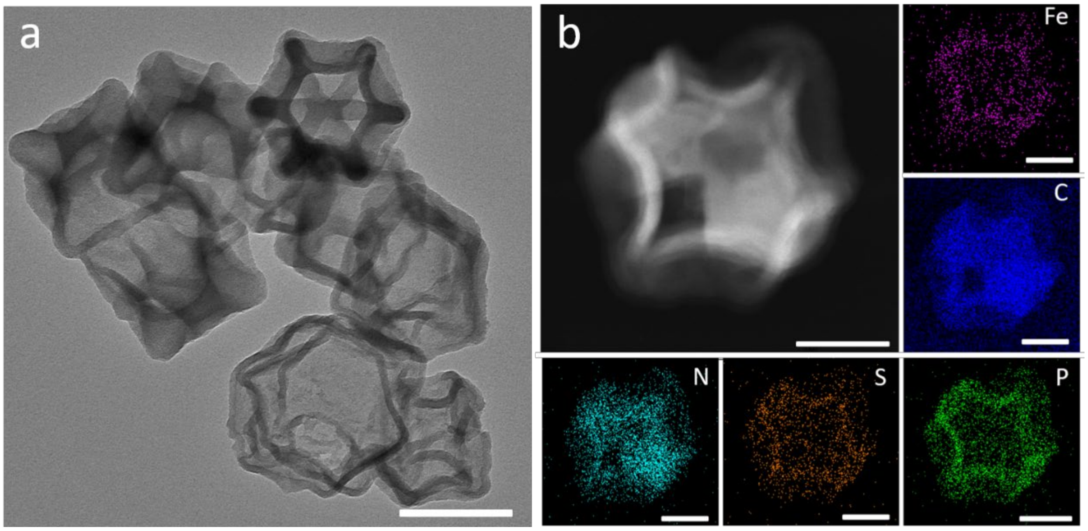

Supplementary Figure 22 | The structural characterizations of the used Fe-SAs/NPS-HC catalyst. a TEM image of the used Fe-SAs/NPS-HC. Scale bar, 500 nm. **b** The enlarged HAADF-STEM image and corresponding EDS element maps (Fe: purple, C: blue, N: cyan, S: orange, P: green). Scale bar, 100 nm.

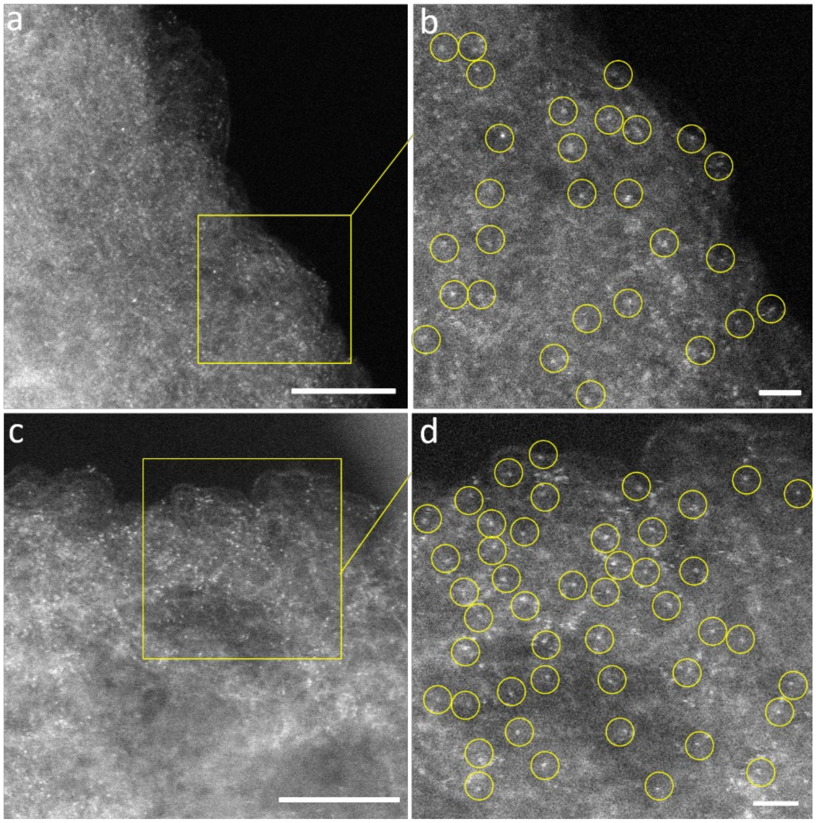

Supplementary Figure 23 | The more aberration-corrected HAADF-STEM images and the enlarged images of the used Fe-SAs/NPS-HC catalyst. The single Fe atoms are marked by yellow circles (**a** and **c**: Scale bar, 5 nm; **b** and **d**: Scale bar, 1 nm).

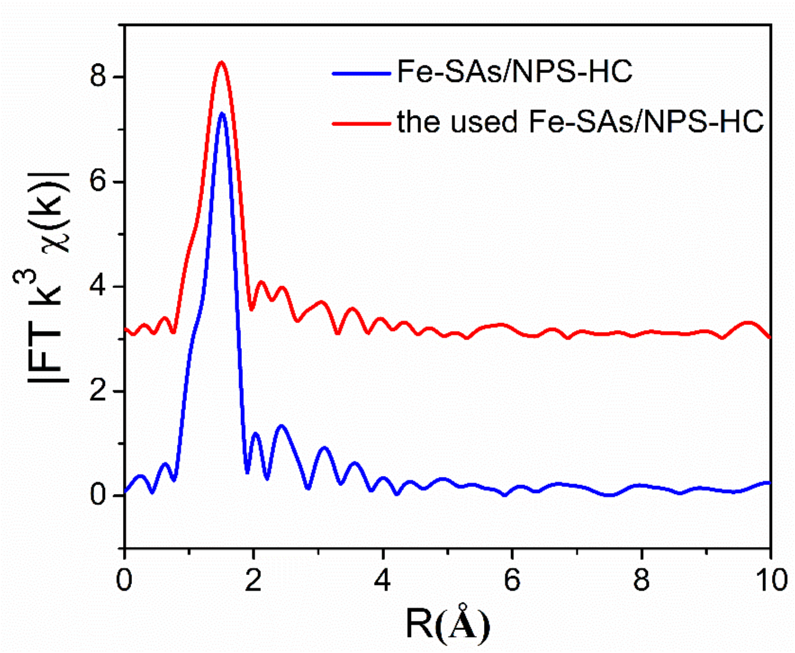

Supplementary Figure 24 | The Fe K-edge k^3-weighted Fourier transform (FT) spectra of the used Fe-SAs/NPS-HC (red line) catalyst and the fresh Fe-SAs/NPS-HC catalyst (blue line).

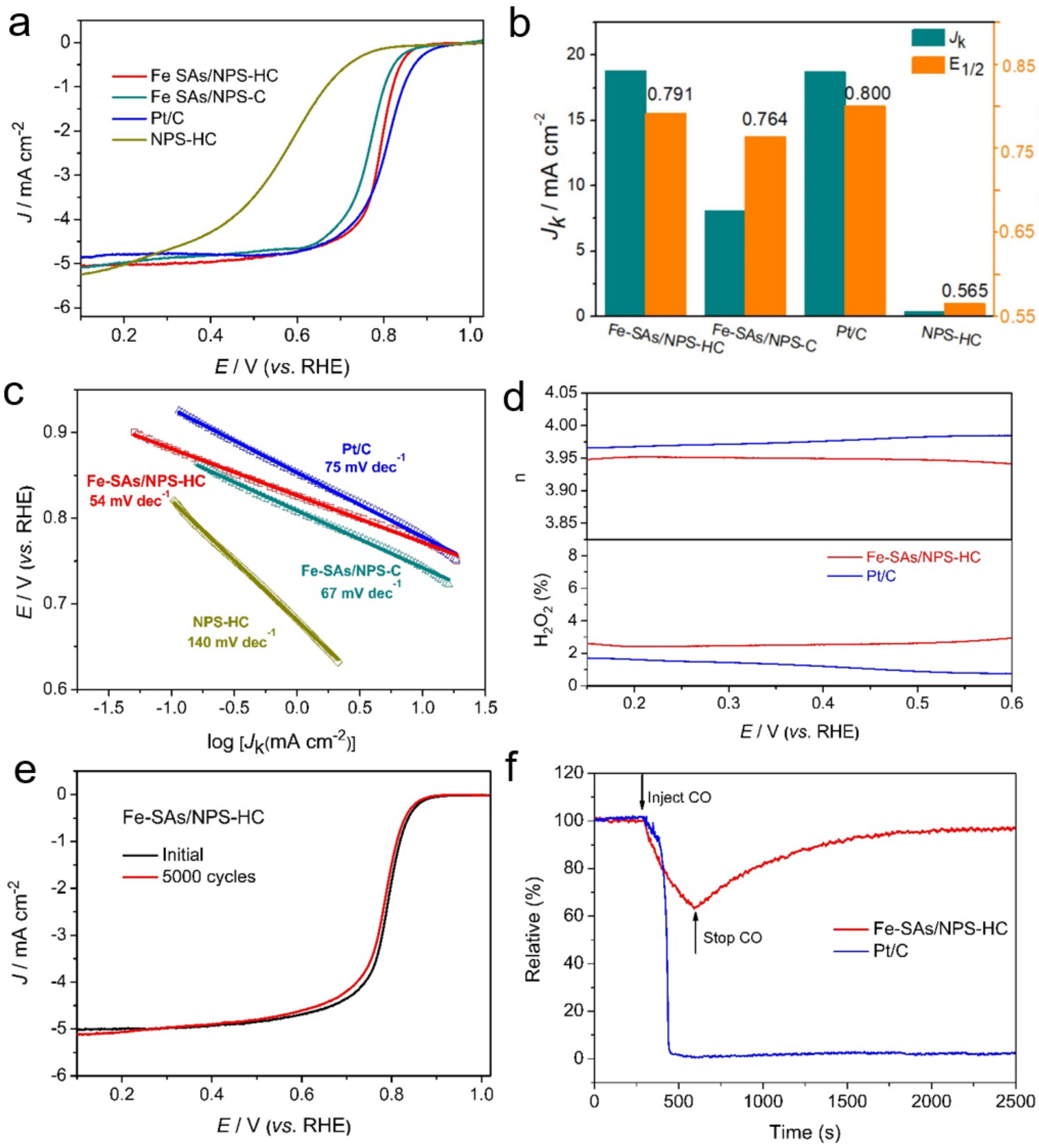

Supplementary Figure 25 | Electrocatalytic ORR performance of Fe-SAs/NPS-HC in 0.5 M H_2SO_4 solution. a ORR polarization curves for Fe-SAs/NPS-HC, Fe-SAs/NPS-C, NPS-HC and 20% Pt/C. **b** Comparison of J_k at 0.75 V and $E_{1/2}$ of Fe-SAs/NPS-HC and the corresponding reference catalysts. **c** The Tafel plots for Fe-SAs/NPS-HC and the corresponding reference catalysts. **d** Electron transfer number (n) (top) and H_2O_2 yield (bottom) versus potential. **e** ORR polarization curves of Fe-SAs/NPS-HC before and after 5000 potential cycles. **f** Tolerance to CO poison of Fe-SAs/NPS-HC compared with 20% Pt/C at 0.45 V (vs. RHE). CO (flow 50 mL·s^{-1}) is injected into the 0.5 M H_2SO_4 solution at the time of 300 s and stopped at the time of 600 s.

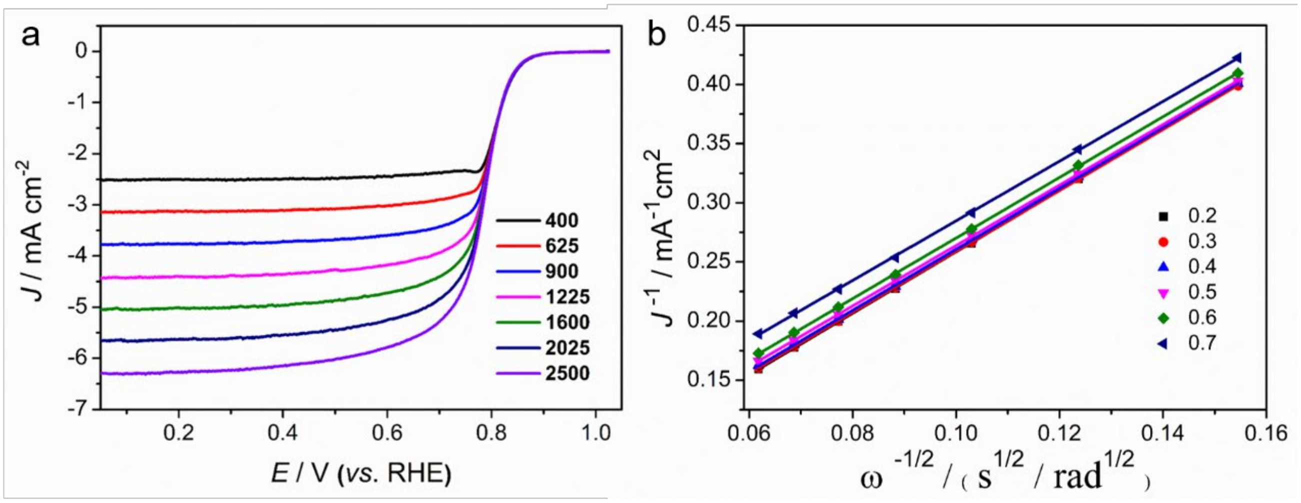

Supplementary Figure 26 | Electrocatalytic ORR performance of Fe-SAs/NPS-HC in 0.5 M H_2SO_4 solution. a The ORR polarization curves at different rotating rates of Fe-SAs/NPS-HC. **b** K-L plots and electron transfer numbers.

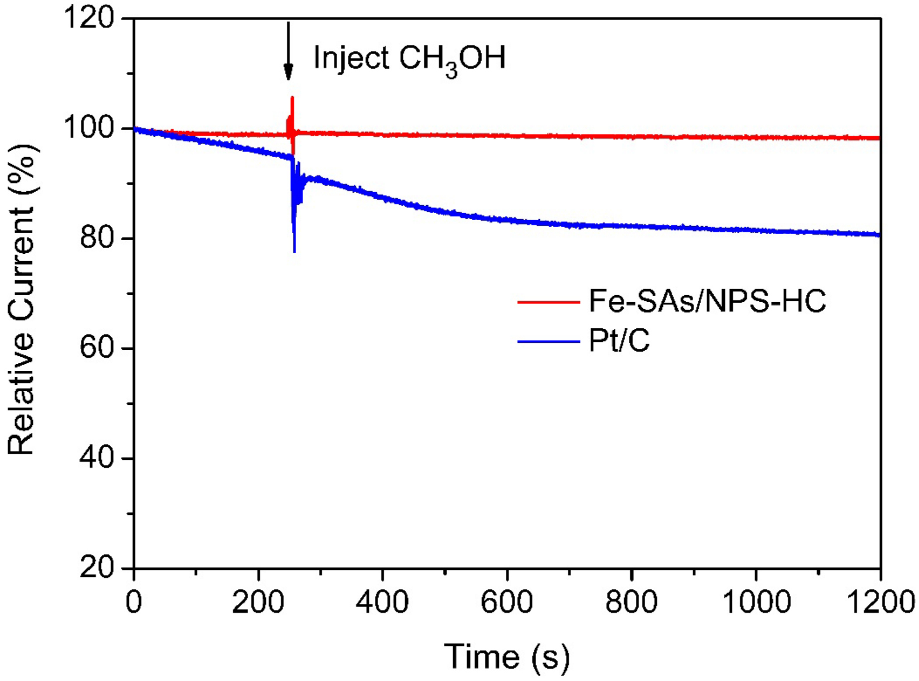

Supplementary Figure 27 | Tolerance to methanol of Fe-SAs/NPS-HC compared with the 20% Pt/C at 0.45 V (vs. RHE) in 0.5 M H_2SO_4 solution.

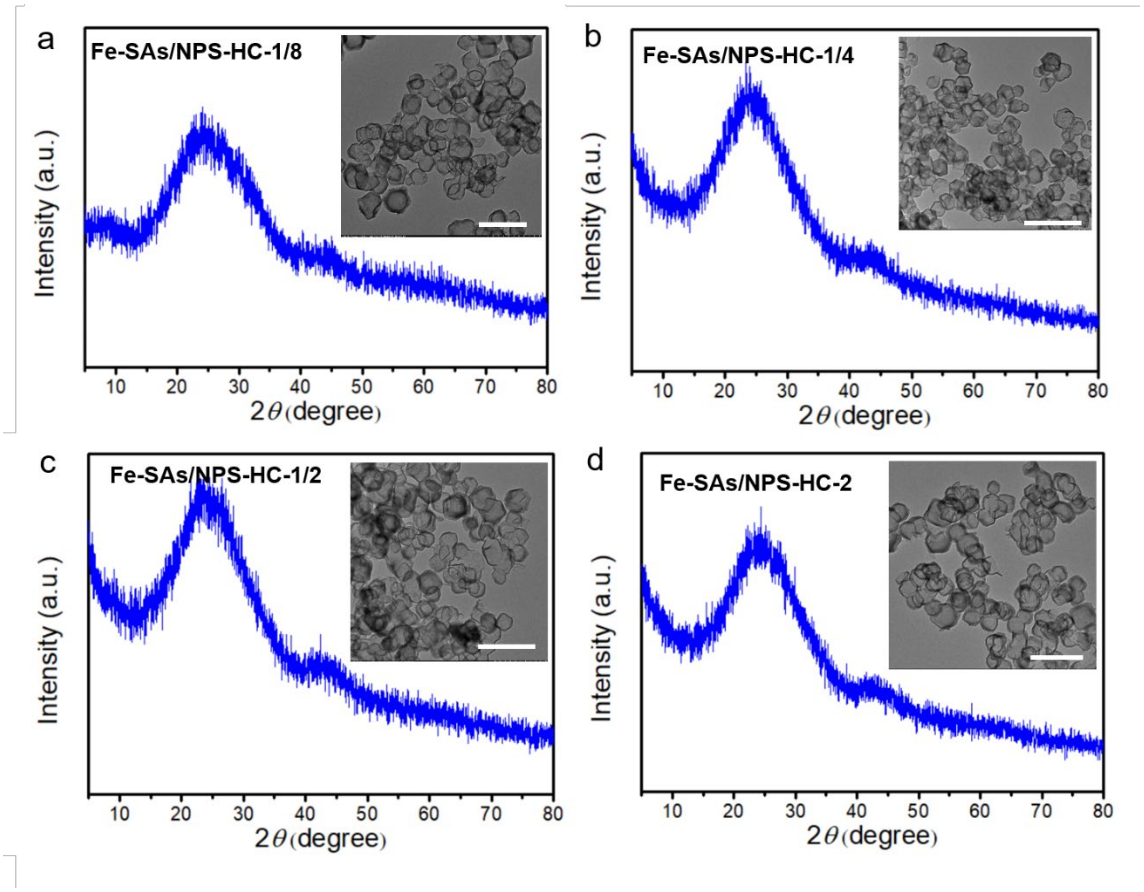

Supplementary Figure 28 | XRD patterns of the Fe-SAs/NPS-HC samples with different Fe loadings. The synthesis of these Fe-SAs/NPS-HC samples with different Fe loadings are the same as that of Fe-SAs/NPS-HC expect that the one-eighth of iron precursor, one-quarter of iron precursor, half of iron precursor and double iron precursor are used, respectively, which are denoted as Fe-SAs/NPS-HC-1/8, Fe-SAs/NPS-HC-1/4, Fe-SAs/NPS-HC-1/2 and Fe-SAs/NPS-HC-2. **a** Fe-SAs/NPS-HC-1/8, **b** Fe-SAs/NPS-HC-1/4, **c** Fe-SAs/NPS-HC-1/2 and **d** Fe-SAs/NPS-HC-2. Inset: The corresponding TEM images. Scale bar, 1 μm.

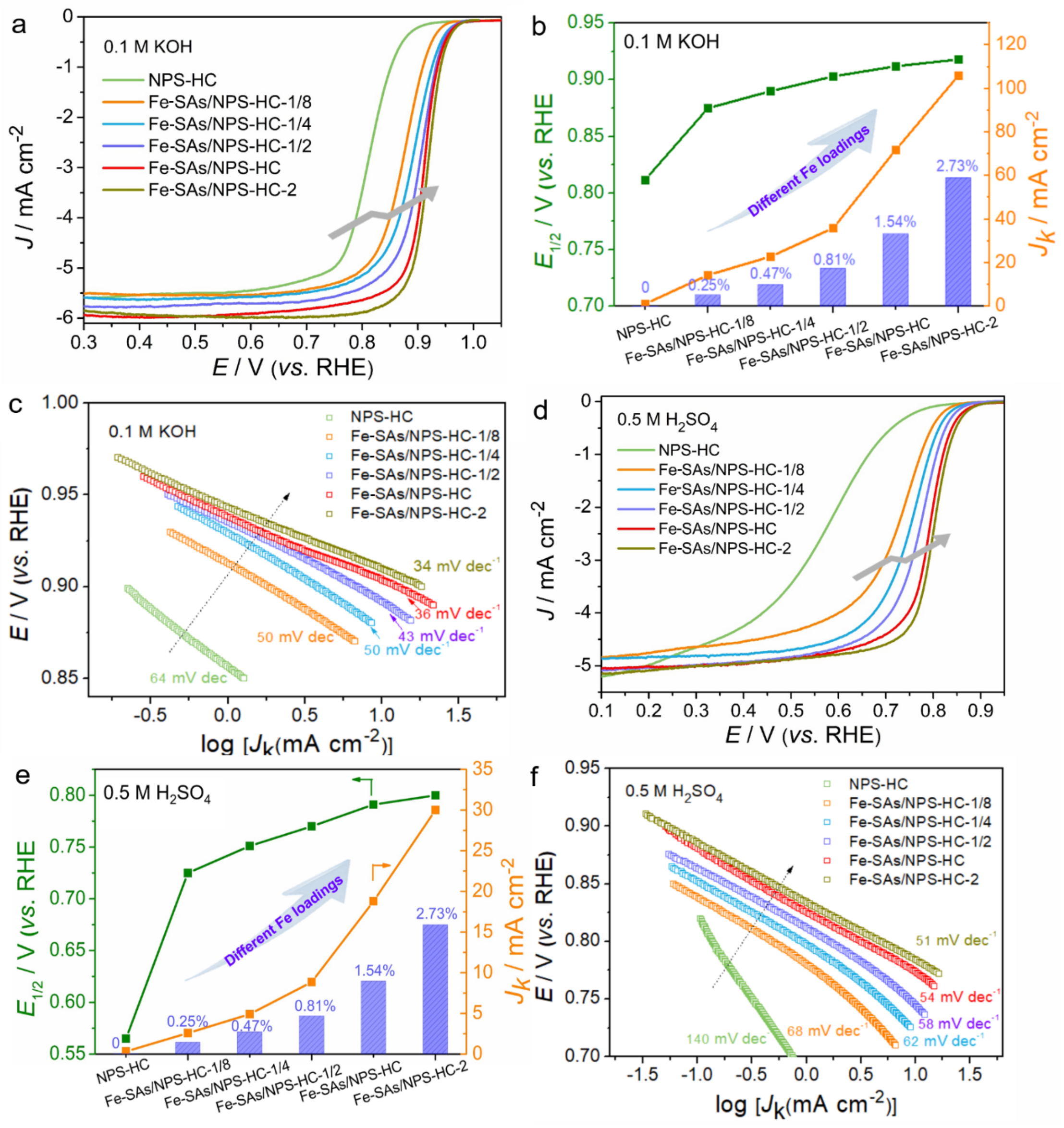

Supplementary Figure 29 | Electrocatalytic ORR performance of Fe-SAs/NPS-HC samples with different Fe loadings. a-c ORR polarization curves (**a**), comparrison of J_k at 0.85 V and $E_{1/2}$ (**b**), Tafel plots (**c**) of NPS-HC, Fe-SAs/NPS-HC-1/8, Fe-SAs/NPS-HC-1/4, Fe-SAs/NPS-HC-1/2, Fe-SAs/NPS-HC, Fe-SAs/NPS-HC-2 in alkaline media. **d-f** ORR polarization curves (**d**), comparrison of J_k at 0.85 V and $E_{1/2}$ (**e**), Tafel plots (**f**) of NPS-HC, Fe-SAs/NPS-HC-1/8, Fe-SAs/NPS-HC-1/4, Fe-SAs/NPS-HC-1/2, Fe-SAs/NPS-HC, Fe-SAs/NPS-HC-2 in acidic media. The content of Fe is determined by ICP-OES as 0, 0.25 wt%, 0.47 wt%, 0.81 wt%, 1.54 wt% and 2.73 wt% for NPS-HC, Fe-SAs/NPS-HC-1/8, Fe-SAs/NPS-HC-1/4, Fe-SAs/NPS-HC-1/2, Fe-SAs/NPS-HC and Fe-SAs/NPS-HC-2, respectively.

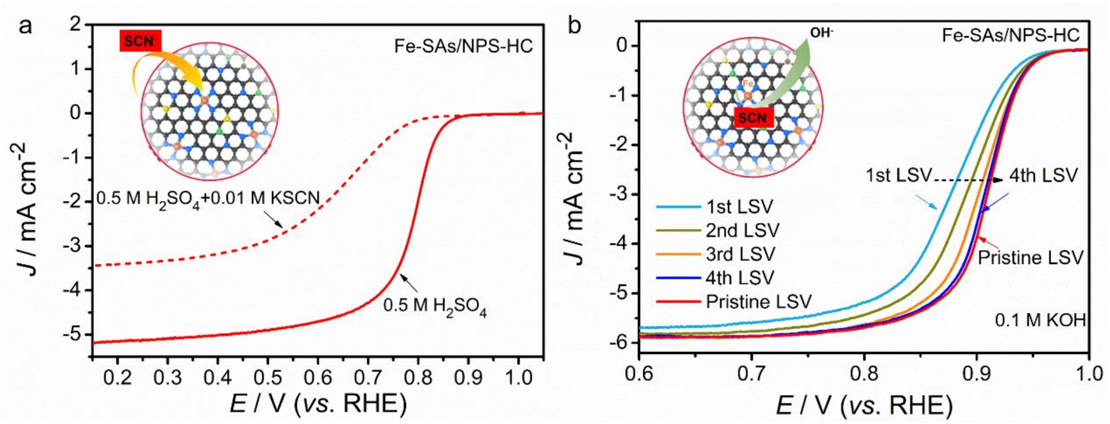

Supplementary Figure 30 | a ORR polarization curves of Fe-SAs/NPS-HC before and after the addition of 0.01 M KSCN in 0.5 M H_2SO_4. **b** ORR polarization curves of SCN^- poisoned Fe-SAs/NPS-HC by continuous LSV tests in 0.1 M KOH.

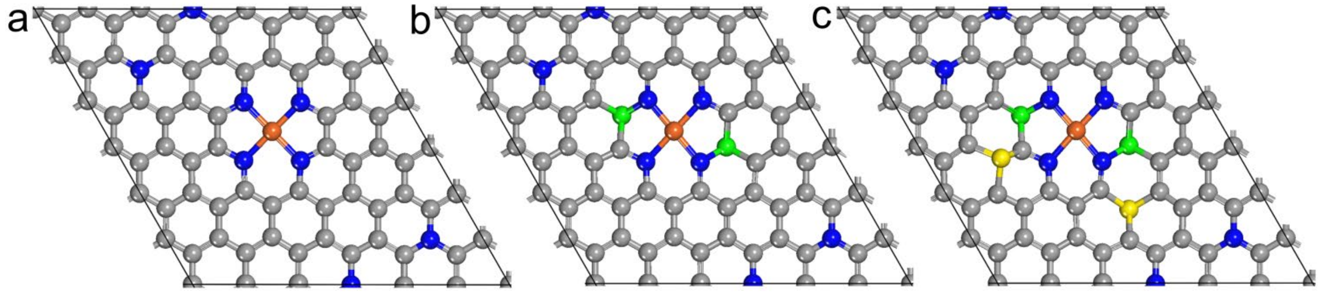

Supplementary Figure 31 | Computational models of different doped types of Fe-SAs samples. a Fe-SAs/N-C, **b** Fe-SAs/NP-C and **c** Fe-SAs/NPS-C (C: gray, N: blue, Fe: orange, P: green, S: yellow).

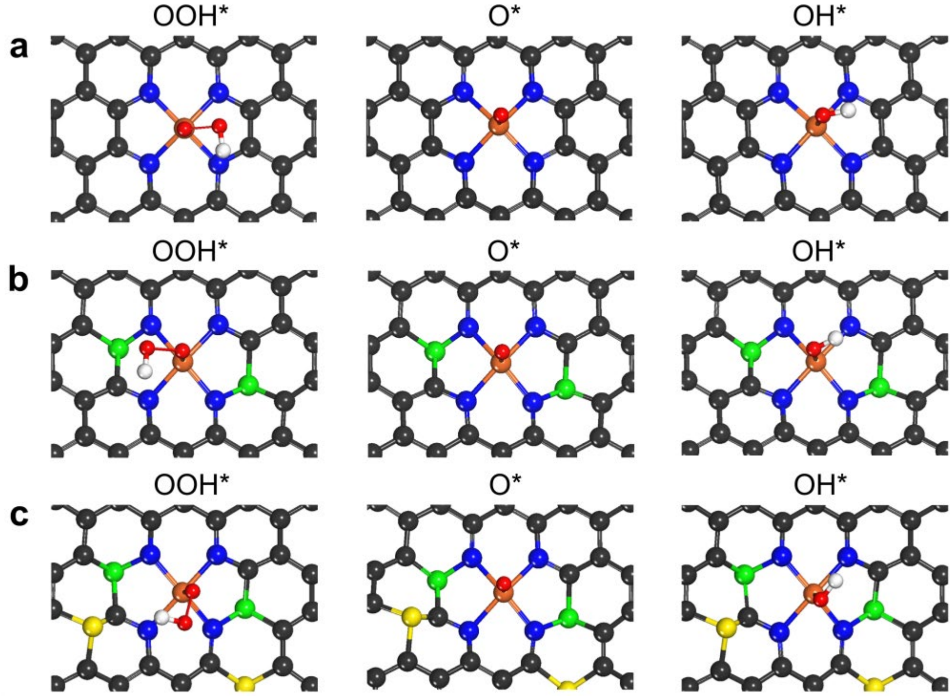

Supplementary Figure 32 | Configurations of adsorbates on different doped types of Fe-SAs samples. a Fe-SAs/N-C, **b** Fe-SAs/NP-C and **c** Fe-SAs/NPS-C (C: black, N: blue, Fe: orange, P: green, S: yellow, O: red, H: white).

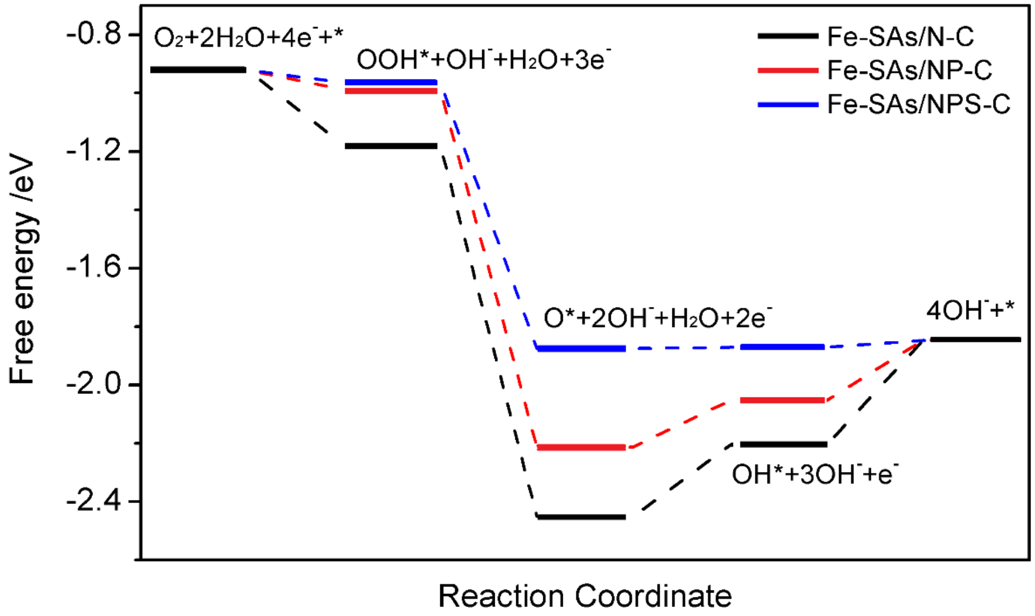

Supplementary Figure 33 | Free energy diagram of ORR on different doped types of Fe-SAs samples at U^{RHE} = 1.0 V. The last two electrochemical steps of Fe-SAs/N-C and Fe-SAs/NP-C are relatively strong endothermic at U^{RHE} = 1.0 V, suggesting that Fe-SAs/N-C and Fe-SAs/NP-C exhibit less reactivity than Fe-SAs/NPS-C. ($U^{RHE} = U^{NHE} + 0.0591pH$).

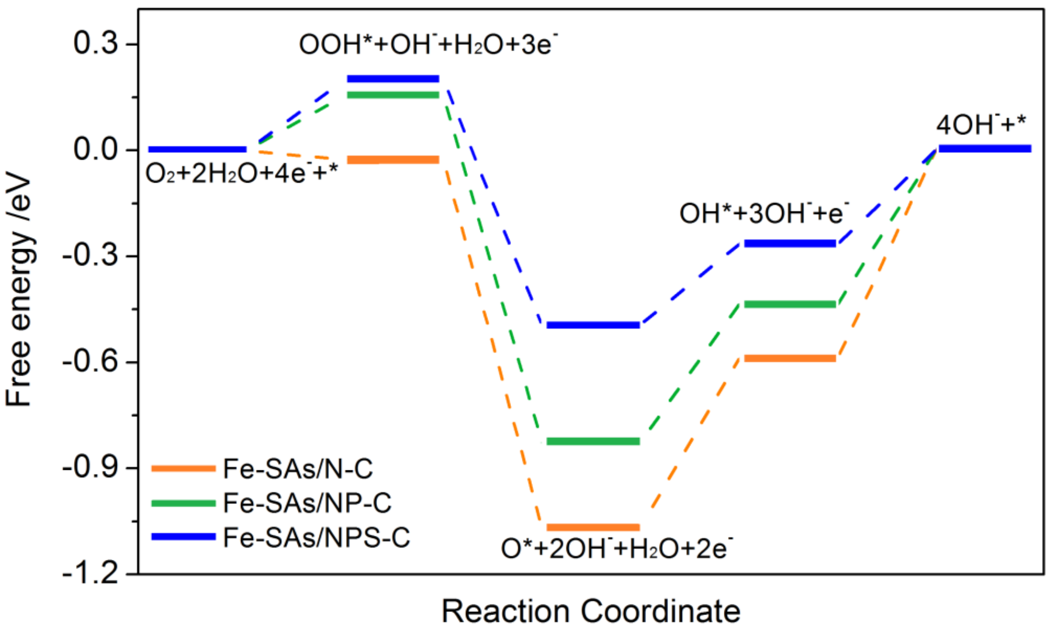

Supplementary Figure 34 | Free energy diagram of ORR at U^{RHE} = 1.23 V on different doped types of Fe-SAs/NPS-C, Fe-SAs/N-C and Fe-SAs/NP-C. The last two electrochemical steps of Fe-SAs/N-C and Fe-SAs/NP-C are more endothermic than Fe-SAs/NPS-C at U^{RHE} = 1.23 V, suggesting that Fe-SAs/N-C and Fe-SAs/NP-C exhibit less reactivity than Fe-SAs/NPS-C.

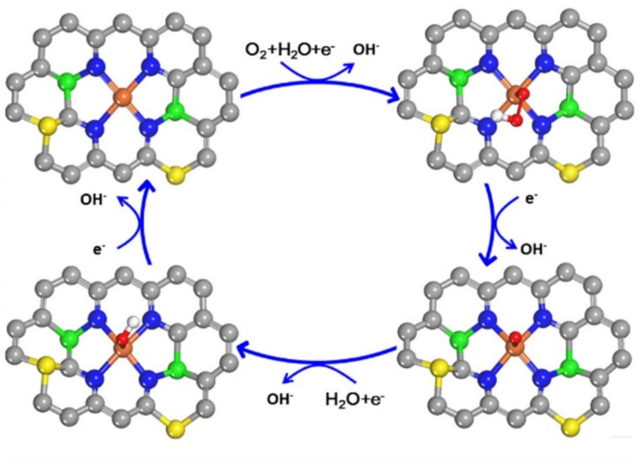

Supplementary Figure 35 | Proposed reaction scheme with the intermediates having optimized geometry of Fe-SAs/NPS-HC toward alkaline ORR. (C: grey, N: blue, Fe: orange, P: green, S: yellow, O: red, H: white)

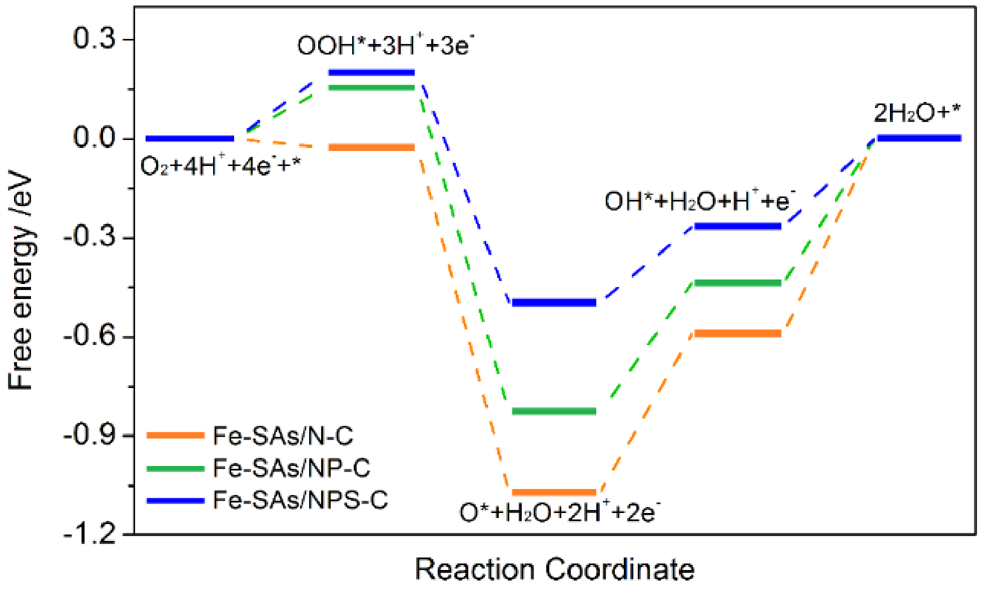

Supplementary Figure 36 | Free energy diagram of ORR on different doped types of Fe-SAs samples at U^{RHE} = 1.23 V under acid condition. The last two electrochemical steps of Fe-SAs/N-C and Fe-SAs/NP-C are relatively strong endothermic, suggesting that Fe-SAs/N-C and Fe-SAs/NP-C exhibit less reactivity than Fe-SAs/NPS-C.

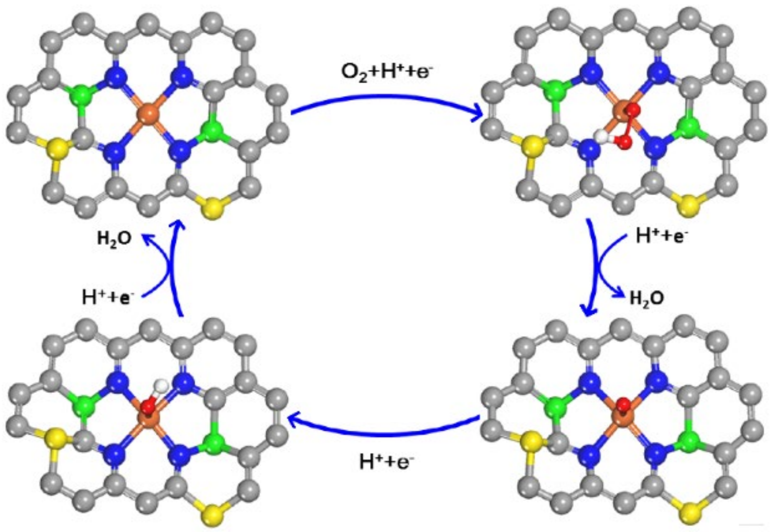

Supplementary Figure 37 | Proposed reaction scheme with the intermediates having optimized geometry of Fe-SAs/NPS-HC toward acidic ORR. (C: grey, N: blue, Fe: orange, P: green, S: yellow, O: red, H: white)

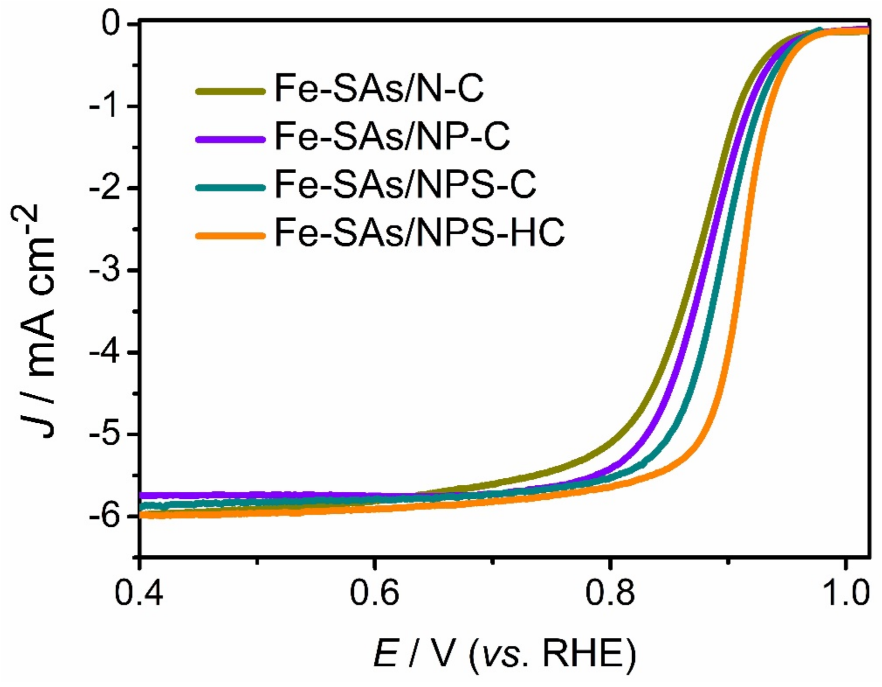

Supplementary Figure 38 | ORR polarization curves of single-atom Fe catalysts with different doped types.

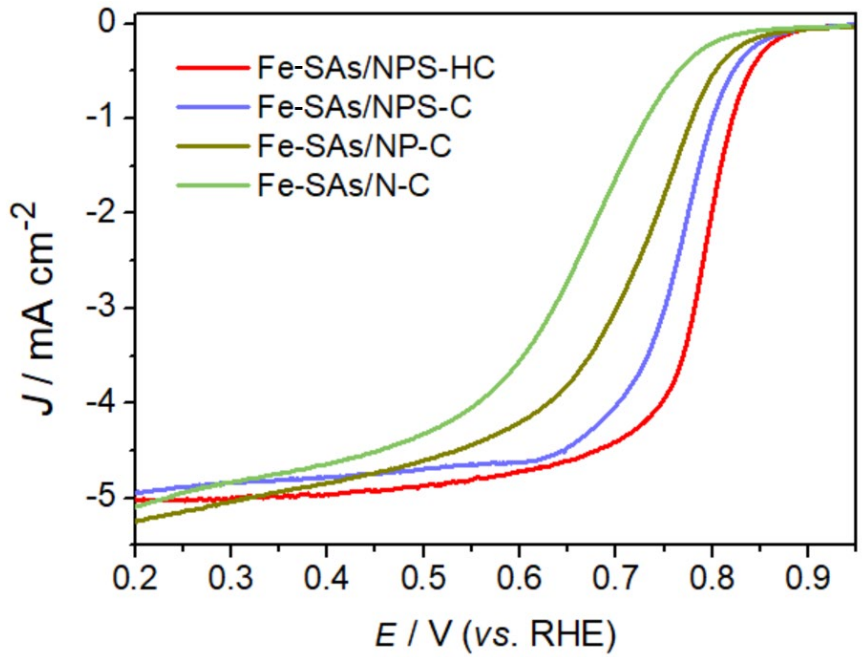

Supplementary Figure 39 | ORR polarization curves of single-atom Fe catalysts with different doped types in O_2-saturated 0.5 M H_2SO_4.

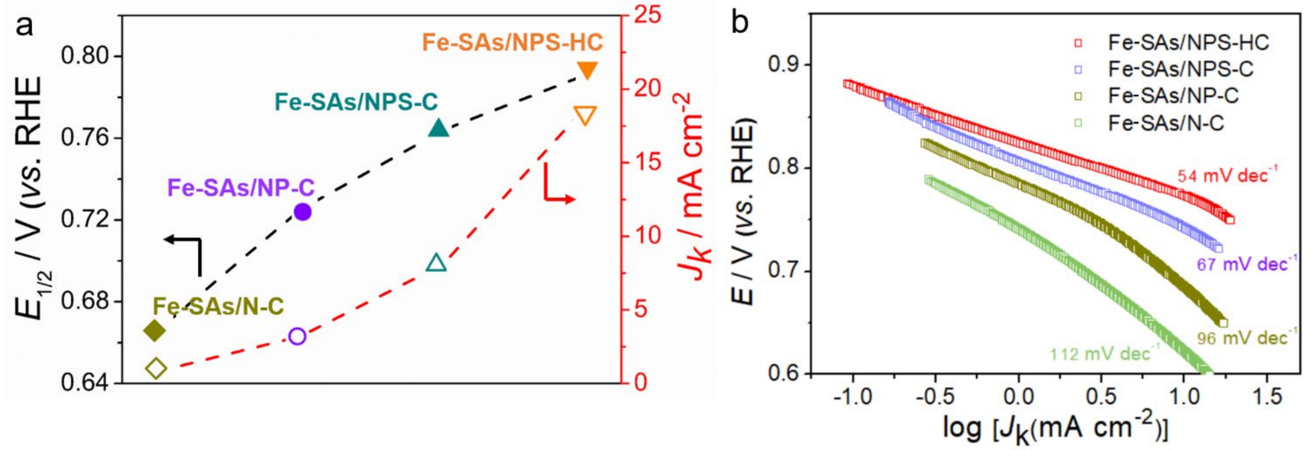

Supplementary Figure 40 | Electronic control effect on acidic ORR performance by experimental measurements.

a comparison of J_k at 0.85 V and and $E_{1/2}$ and **b** the Tafel plots of single-atom Fe catalysts with different doped types.

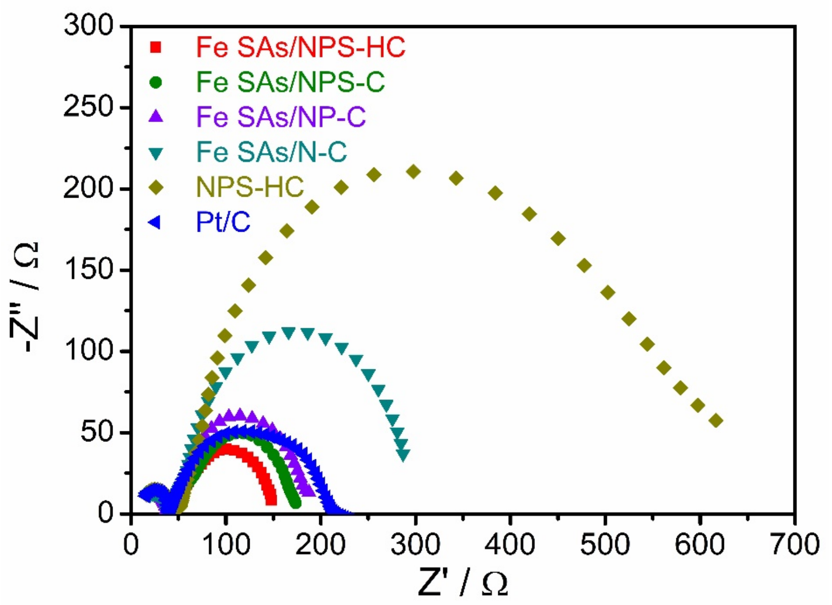

Supplementary Figure 41 | Nyquist plots of Fe-SAs/NPS-HC and the corresponding reference catalysts in 0.1 M KOH.

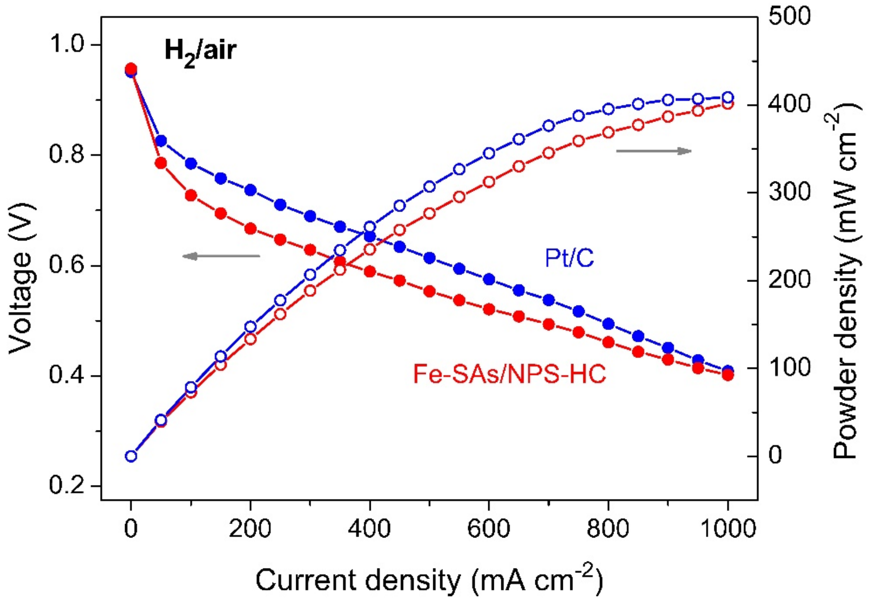

Supplementary Figure 42| H_2/air fuel cell performance of Fe-SAs/NPS-HC at 80 °C. H_2/air fuel cell polarization curves and power density plots of MEAs using Fe-SAs/NPS-HC (loading of 0.8 mg cm^{-2}) and 20 wt% Pt/C (loading of 0.8 mg cm^{-2}) as cathode catalysts, respectively. Membrane Nafion 211, cell 80 °C, electrode area 5 cm^2.

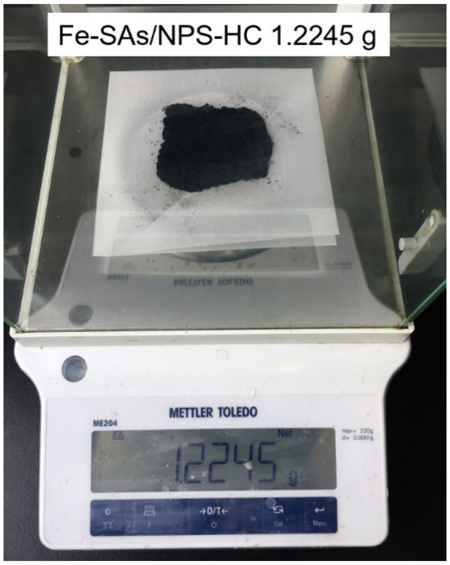

Supplementary Figure 43 | Picture of ~1.22 g Fe-SAs/NPS-HC catalyst.

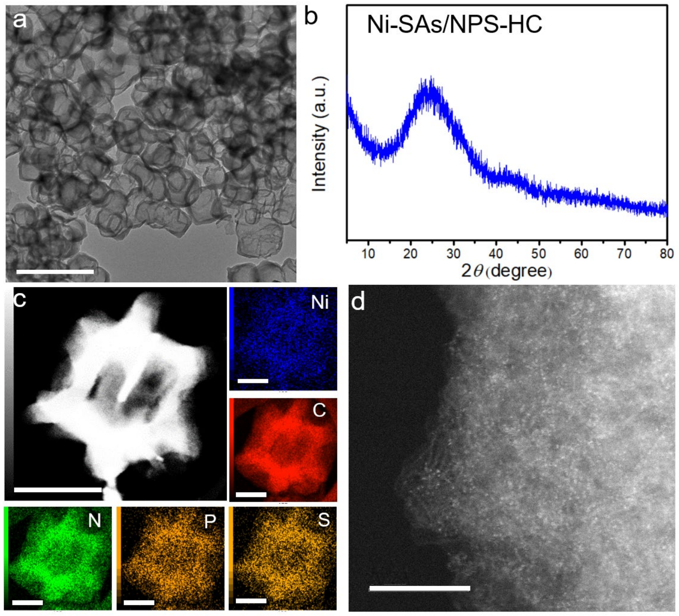

Supplementary Figure 44 | Characterizations of the Ni-SAs/NPS-HC catalyst. a TEM image of the Ni-SAs/NPS-HC. Scale bar, 500 nm. **b** XRD pattern of the Ni-SAs/NPS-HC catalyst. **c** HAADF-STEM image and corresponding EDS element maps (Ni: blue, C: red, N: green, P: orange, S: yellow). Scale bar, 100 nm. **d** AC HAADF-STEM image shows that isolated Ni single atoms are observed. Scale bar, 5 nm.

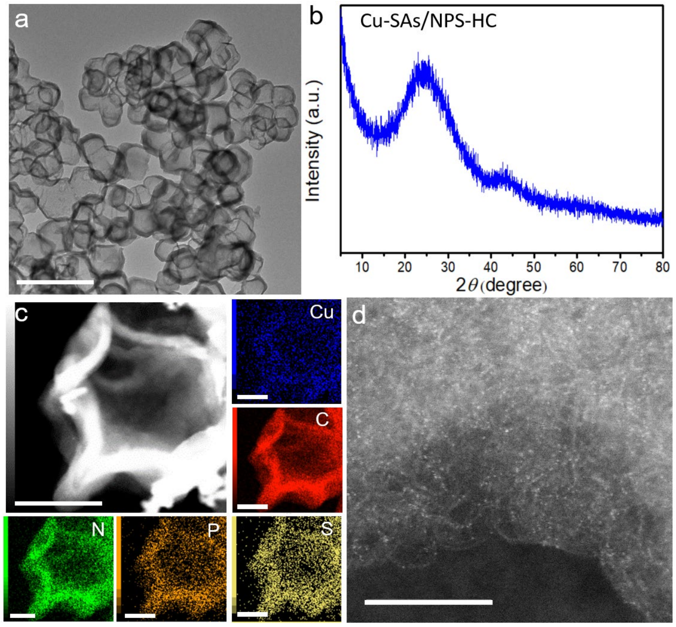

Supplementary Figure 45 | Characterizations of the Cu-SAs/NPS-HC catalyst. a TEM image of the Cu-SAs/NPS-HC. Scale bar, 500 nm. **b** XRD pattern of the Cu-SAs/NPS-HC catalyst. **c** HAADF-STEM image and corresponding EDS element maps (Cu: blue, C: red, N: green, P: orange, S: yellow). Scale bar, 100 nm. **d** AC HAADF-STEM image shows that isolated Cu single atoms are observed. Scale bar, 5 nm.

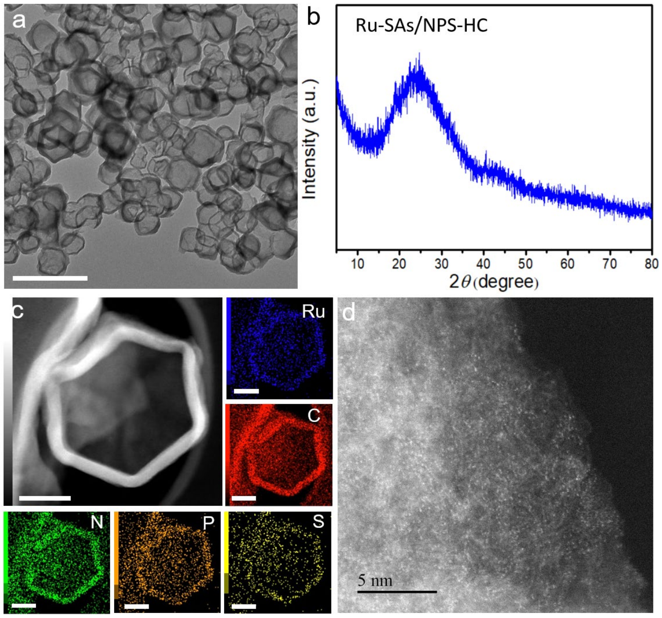

Supplementary Figure 46 | Characterizations of the Ru-SAs/NPS-HC catalyst. a TEM image of the Ru-SAs/NPS-HC. Scale bar, 500 nm. **b** XRD pattern of the Ru-SAs/NPS-HC catalyst. **c** HAADF-STEM image and corresponding EDS element maps (Ru: blue, C: red, N: green, P: orange, S: yellow). Scale bar, 100 nm. **d** AC HAADF-STEM image shows that isolated Ru single atoms are observed. Scale bar, 5 nm.

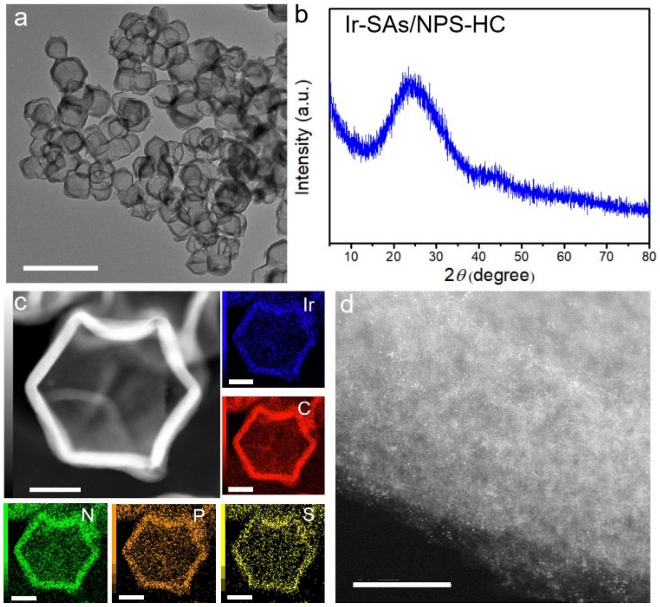

Supplementary Figure 47 | Characterizations of the Ir-SAs/NPS-HC catalyst. a TEM image of the Ir-SAs/NPS-HC. Scale bar, 500 nm. **b** XRD pattern of the Ir-SAs/NPS-HC catalyst. **c** HAADF-STEM image and corresponding EDS element maps (Ir: blue, C: red, N: green, P: orange, S: yellow). Scale bar, 100 nm. **d** AC HAADF-STEM image shows that isolated Ir single atoms are observed. Scale bar, 5 nm.

Supplementary Table 1 | Hyperfine parameters derived from Mössbauer spectra of Fe-SAs/NPS-HC at 292 K and at 78 K: the isomer shifts δ_{iso}, quadrupole splitting ΔE_Q, quadrupole lineshifts ε, internal fields B_{hf}, electric field gradient (EFG) principal components V_{zz}, angles Θ between V_{zz} and B_{hf}, linewidth LW, and subspectra areas.

	T (K)	δ_{iso} (mm/s)	B_{hf}(T)	**ε**** (sites 1, 2) ΔE_Q (site 3) (mm/s)	V_{zz} (10^{21}V/m^2)	Θ (º)	LW (mm/s)	Area (%)	Assignment
Site 1	292	0.41(5)	Relax.*	0.52(0***)	3.11	0	0.26(0)	29	$Fe^{II}N_4$, middle-spin
	78	0.48(2)	17.3(1)	0.52(4)			0.44(5)	31	
Site 2	292	0.40(1)	Relax.*	0.18(0***)	1.05	0	0.26(0)	51	$Fe^{III}N_4$, low-spin
	78	0.45(1)	10.5(1)	0.18(2)			0.46(4)	50	
Site 3	292	0.508(6)	0	0.71(4)	undefined	—	0.26(0)	20	$Fe^{II}N_4$, low-spin
	78	0.54(2)	0	0.90(4)	undefined	—	0.49(6)	19	

Notes: Θ is the polar angle of the hyperfine field B_{hf} in the reference frame of the principal EFG axes (V_{xx},V_{yy}, V_{zz}). Because Θ was fitted to be ≈ 0, the other polar coordinate (angle ϕ) and asymmetry parameter η are undefined, see Eq. (1) below.

*Singlet-shaped collapsed subspectra with slowly decaying wings fitted with magnetic relaxation at the rates of 8 to 8.5 Mrad/s.

** **ε** is the first-order quadrupole lineshift related to the quadrupole energy $\Delta E_Q = e^2qQ/2$ via Θ, ϕ and $\eta = (V_{xx}-V_{yy})/V_{zz}$:

$$\varepsilon = \frac{\Delta E_Q}{4}\left(3\cos^2\theta - 1 + \eta\sin^2\theta\cos 2\phi\right) \tag{1}$$

*** Fixed to be similar to the values obtained at 78K.

Supplementary Table 2 | Structural parameters of Fe-SAs/NPS-HC, Fe foil and Fe_2O_3 extracted from the EXAFS fitting. (S_0^2=0.74)

Sample	Scattering pair	CN	R(Å)	$\sigma^2(10^{-3}Å^2)$	ΔE_0(eV)	R factor
Fe-SAs/NPS-HC	Fe-N1	1.8	2.01	7.8	2.5	0.0026
	Fe-N2	2.1	2.07	8.4		
Fe foil	Fe-Fe1	8*	2.46	4.9	4.5	0.052
	Fe- Fe2	6*	2.84	5.5		
Fe_2O_3	Fe-O1	3*	1.92	4.5	2.7	0.01
	Fe-O2	3*	2.10	6.4		

S_0^2 is the amplitude reduction factor; CN is the coordination number; R is interatomic distance (the bond length between central atoms and surrounding coordination atoms); σ^2 is Debye-Waller factor (a measure of thermal and static disorder in absorber-scatterer distances); ΔE_0 is edge-energy shift (the difference between the zero kinetic energy value of the sample and that of the theoretical model). R factor is used to value the goodness of the fitting.

* This value was fixed during EXAFS fitting, based on the known structure.

Error bounds that characterize the structural parameters obtained by EXAFS spectroscopy were estimated as N ± 20%; R ± 1%; σ^2 ± 20%; ΔE_0 ± 20%. Fe-SAs (FT range: 2.0-11.0 $Å^{-1}$; fitting range: 0.5-2.3 Å) Fe foil (FT range: 2.0-11.0 $Å^{-1}$; fitting range: 1.3-3.0 Å)

Supplementary Table 3 | Comparison of performance for Fe-SAs/NPS-HC in 0.1 M KOH solution, as well as other precious catalysts reported in the literatures.

Electrocatalysts	$E_{1/2}$ (V vs RHE)	Kinetic Current Density (mA cm^{-2})	Tafel (mV $decade^{-1}$)	Ref.
**Fe-SAs/NPS-HC**	**0.912**	**71.9 at 0.85 V**<br>**13.0 at 0.9 V**	**36**	**Our work**
Fe@Aza-PON	0.839	~ 4.3 at 0.85 V	60	1
S,N-Fe/N/C-CNT	0.85	~ 7.4 at 0.85 V	82	2
Fe-ISAs/CN	0.900	37.83 at 0.85 V<br>6.06 at 0.9 V	58	3
pCNT@Fe1.5@GL	0.867	~ 11.5 at 0.85 V	79	4
NC@Co-NGC DSNCs	0.820	12.8 at 0.71 V	51	5
Fe@C-FeNCs-2	0.899	41.6 at 0.8 V	68	6
CNT/PC	0.88	2.4 at 0.9 V	-	7
NCNTFs	0.87	~ 5.8 at 0.8 V	64	8
Co SAs/N-C(900)	0.881	21.2 at 0.8 V	75	9
FePhen@MOF-ArNH3	0.86	2.1 at 0.9 V	-	10
Fe-N/C-800	0.81	0.4 at 0.9 V	59	11
BCNFNHs	0.861	~ 7.0 at 0.8 V	91.2	12
Fe/N/C-SCN	0.836	~ 28 at 0.8 V<br>~ 1.9 at 0.85 V	66	13
Fe/N/C-Cl	0.820	~ 9.3 at 0.8 V	64	13
Meso/micro-PoPD	0.85	~ 10 at 0.8 V	-	14
N-doped C/CNTs	0.82	8.30 at 0.8 V	65.1~68.1	15
NT-G	0.87	2.8 at 0.9 V	-	16

Supplementary Table 4 | Comparison of performance for Fe-SAs/NPS-HC and other non-noble catalysts reported in the literatures in 0.5 M H_2SO_4.

Electrocatalysts	Rotation and scan rate	$E_{1/2}$ (V vs. RHE)	current density (mA cm^{-2}) at 0.75 V	Kinetic Current Density (mA cm^{-2}) 0.75 V	Limited current	Ref.
**Fe-SAs/NPS-HC**	**900 rpm and 10 mVs^{-1}**	**0.801**	**3.21**	**21.9**	**3.76**	**Our work**
	**1600 rpm and 10 mV s^{-1}**	**0.791**	**3.95**	**18.8**	**5.01**	
PANI-FeCo-C	900 rpm and 10 mVs^{-1}	0.805	2.9	11.3	3.9	17
N-Fe-MOF	900 rpm and 10 mVs^{-1}	0.79	2.4	6.67	3.75	18
Fe-CNT-PA1600	1600 rpm and 5 mV s^{-1}	0.65	1.1	1.35	5.9	19
Co-N-GA	1600 rpm and 10 mV s^{-1}	0.73	2.5	4.39	5.8	20
Fe-N-CC	1600 rpm and 10 mV s^{-1}	0.62	0.5	0.583	3.5	21
ISAS-Co/HNCS	1600 rpm and 10 mV s^{-1}	0.773	3.9	12.8	5.61	22
(CM+PANI)-Fe-C	900 rpm and 10 mVs^{-1}	0.8	2.6	19.5	3.0	23
SA-Fe/NG	1600 rpm and 5 mV s^{-1}	0.8	3.9	17.7	5.0	24

Supplementary Table 5 | The Fe content of Fe-based catalysts determined by ICP-OES analysis.

Catalysts	Fe-SAs/NPS-HC-1/8	Fe-SAs/NPS-HC-1/4	Fe-SAs/NPS-HC-1/2	Fe-SAs/NPS-HC	Fe-SAs/NPS-HC-2
Fe content (wt%)	0.25	0.47	0.81	1.54	2.73

Notes: The synthesis of these Fe-SAs/NPS-HC samples with different Fe loadings are the same as that of Fe-SAs/NPS-HC expect that the one-eighth of iron precursor, one-quarter of iron precursor, half of iron precursor and double iron precursor are used, respectively, which are denoted as Fe-SAs/NPS-HC-1/8, Fe-SAs/NPS-HC-1/4, Fe-SAs/NPS-HC-1/2 and Fe-SAs/NPS-HC-2.

Supplementary Table 6 | Calculated Gibbs free energies (eV) of ORR elementary steps under reaction condition (T = 298 K, U^{NHE} = 0.23 V, pH = 13) on Fe-SAs/N-C.

Elementary steps	ΔE	ΔH	ΔZPE	$T\Delta S$	ΔG_0	ΔG
$O_2(g) + H_2O(l) + 4e^- + * \rightarrow OOH* + OH^- + 3e^-$	-2.14	-0.13	0.19	-0.84	-1.26	-0.26
$OOH* + 3e^- \rightarrow O* + OH^- + 2e^-$	-1.94	0.06	0.08	0.47	-2.27	-1.27
$O* + H_2O(l) + 2e^- \rightarrow OH* + OH^- + e^-$	-1.05	-0.04	0.14	-0.20	-0.75	0.24
$OH* + e^- \rightarrow OH^- + *$	-0.32	0.06	0.09	0.47	-0.64	0.36

Supplementary Table 7 | Calculated Gibbs free energies (eV) of ORR elementary steps under reaction condition (T = 298 K, U^{NHE} = 0.23 V, pH = 13) on Fe-SAs/NP-C.

Elementary steps	ΔE	ΔH	ΔZPE	$T\Delta S$	ΔG_0	ΔG
$O_2(g) + H_2O(l) + 4e^- + * \rightarrow OOH^* + OH^- + 3e^-$	-1.96	-0.13	0.19	-0.84	-1.07	-0.07
$OOH^* + 3e^- \rightarrow O^* + OH^- + 2e^-$	-1.88	0.06	0.07	0.47	-2.22	-1.22
$O^* + H_2O(l) + 2e^- \rightarrow OH^* + OH^- + e^-$	-1.14	-0.04	0.14	-0.20	-0.84	0.16
$OH^* + e^- \rightarrow OH^- + *$	-0.47	0.06	0.09	0.47	-0.79	0.21

Supplementary Table 8 | Calculated Gibbs free energies (eV) of ORR elementary steps under reaction condition (T = 298 K, U^{NHE} = 0.23 V, pH = 13) on Fe-SAs/NPS-C.

Elementary steps	ΔE	ΔH	ΔZPE	$T\Delta S$	ΔG_0	ΔG
$O_2(g) + H_2O(l) + 4e^- + * \rightarrow OOH* + OH^- + 3e^-$	-1.93	-0.13	0.18	-0.84	-1.04	-0.04
$OOH* + 3e^- \rightarrow O* + OH^- + 2e^-$	-1.58	0.06	0.08	0.47	-1.91	-0.91
$O* + H_2O(l) + 2e^- \rightarrow OH* + OH^- + e^-$	-1.30	-0.04	0.14	-0.20	-0.99	0.00
$OH* + e^- \rightarrow OH^- + *$	-0.65	0.06	0.09	0.47	-0.97	0.02

Supplementary Table 9 | Comparison of mass activity and turnover frequency of Fe-SAs/NPS-HC with other non-noble single-atom catalysts reported in literatures.

Electrocatalysts	$E_{1/2}$ (V vs RHE)	Kinetic Current Density at 0.85 V (mA cm^{-2})	MA at 0.85 V (A/g)*10^{-3}	TOF [e $(site•s)^{-1}$]	Ref.
**Fe-SAs/NPS-HC**	**0.912**	**71.9**	**9.15**	**5.29**	**Our work**
**Fe-SAs/NPS-C**	**0.894**	**34.6**	**4.4**	**2.54**	**Our work**
**Fe-SAs/NP-C**	**0.881**	**19.7**	**2.51**	**1.45**	**Our work**
**Fe-SAs/N-C**	**0.87**	**11.7**	**1.49**	**0.861**	**Our work**
CNT/PC (Fe)	0.88	25.7	1.1	0.638	7
S,N-Fe/N/C-CNT	0.85	7.35	0.368	0.213	2
Fe-ISAs/CN	0.9	37.83	4.29	2.48	3
Fe-N4 SAs/NPC	0.885	7.47	0.747	0.432	25
FeSA-N-C	0.891	23.27	4.63	2.68	26
Co SAs/N-C(900)	0.881	15.3	0.937	0.572	9
Co-ISAS/p-CN	0.838	2.92	1.36	0.83	27
Cu-N-C-60	0.8	1.32	0.0507	0.0334	28

Supplementary Table 10 | Comparison of H_2/air fuel cell performance by using Fe-SAs/NPS-HC and other non-noble catalysts reported in the literatures as the cathode

Catalysts	Catalyst loading (mg cm^{-2})	Back pressure (MPa)	Operation temperature (°C)	Peak power density (mW cm^{-2})	Ref.
**Fe-SAs/NPS-HC**	**0.8**	**0.2**	**60**	**333**	**Our work**
		**0.2**	**80**	**400**	
(CM+PANI)-Fe-C	4	0.1	80	420	23
Fe-N-C-Phen-PANI	4	0.138	80	380	29
Fe-P-C_Ar-NH900	4	0.138	80	335	30
Fe-MBZ	3.0 ± 0.5	0.4	80	330	31
FePhen@MOF-ArNH$_3$	3	0.25	80	380	10
Fe/oPD–Mela	3	0.20	80	270	32
Fe/PI-1000-III-NH3	4	0.2	80	320	33
Fe/TPTZ/ZIF-8	1.14	0.1	80	300	34
Co-PPY-C	0.06 (Co loading)	0.2	80	70	35

Supplementary References

1 Kim, S.-J. *et al.* Defect-free encapsulation of Fe0 in 2D fused organic networks as a durable oxygen reduction electrocatalyst. *J. Am. Chem. Soc.* **140,** 1737-1742 (2018).

2 Chen, P. *et al.* Atomically dispersed iron–nitrogen species as electrocatalysts for bifunctional oxygen evolution and reduction reactions. *Angew. Chem. Int. Ed.* **56,** 610-614 (2017).

3 Chen, Y. *et al.* Isolated single iron atoms anchored on N-doped porous carbon as an efficient electrocatalyst for the oxygen reduction reaction. *Angew. Chem. Int. Ed.* **56,** 6937-6941 (2017).

4 Ahn, S. H., Yu, X. & Manthiram, A. "Wiring" Fe-Nx-embedded porous carbon framework onto 1D nanotubes for efficient oxygen reduction reaction in alkaline and acidic media. *Adv. Mater.* **29,** 1606534 (2017).

5 Liu, S. *et al.* Metal–organic-framework-derived hybrid carbon nanocages as a bifunctional electrocatalyst for oxygen reduction and evolution. *Adv. Mater.* **29,** 1700874 (2017).

6 Jiang, W.-J. *et al.* Understanding the high activity of Fe–N–C electrocatalysts in oxygen reduction: Fe/Fe_3C nanoparticles boost the activity of Fe–Nx. *J. Am. Chem. Soc.* **138,** 3570-3578 (2016).

7 Sa, Y. J. *et al.* A general approach to preferential formation of active Fe–Nx sites in Fe–N/C electrocatalysts for efficient oxygen reduction reaction. *J. Am. Chem. Soc.* **138,** 15046-15056 (2016).

8 Xia, B. Y. *et al.* A metal–organic framework-derived bifunctional oxygen electrocatalyst. *Nat. Energy* **1,** 15006 (2016).

9 Yin, P. *et al.* Single cobalt atoms with precise N-coordination as superior oxygen reduction reaction catalysts. *Angew. Chem. Int. Ed.* **55,** 10800-10805 (2016).

10 Strickland, K. *et al.* Highly active oxygen reduction non-platinum group metal electrocatalyst without direct metal–nitrogen coordination. *Nat. Commun.* **6,** 7343 (2015).

11 Niu, W. *et al.* Mesoporous N-doped carbons prepared with thermally removable nanoparticle templates: an efficient electrocatalyst for oxygen reduction reaction. *J. Am. Chem. Soc.* **137,** 5555-5562 (2015).

12 Yang, W., Liu, X., Yue, X., Jia, J. & Guo, S. Bamboo-like carbon nanotube/Fe_3C nanoparticle hybrids and their highly efficient catalysis for oxygen reduction. *J. Am. Chem. Soc.* **137,** 1436-1439 (2015).

13 Wang, Y.-C. *et al.* S-doping of an Fe/N/C ORR catalyst for polymer electrolyte membrane fuel cells with high power density. *Angew. Chem. Int. Ed.* **54,** 9907-9910 (2015).

14 Liang, H.-W., Zhuang, X., Brüller, S., Feng, X. & Müllen, K. Hierarchically porous carbons with optimized nitrogen doping as highly active electrocatalysts for oxygen reduction. *Nat. Commun.* **5,** 4973 (2014).

15 Sa, Y. J. *et al.* Carbon nanotubes/heteroatom-doped carbon core–sheath nanostructures as highly active, metal-free oxygen reduction electrocatalysts for alkaline fuel cells. *Angew. Chem. Int. Ed.* **53,** 4102-4106 (2014).

16 Li, Y. *et al.* An oxygen reduction electrocatalyst based on carbon nanotube–graphene complexes. *Nat. Nanotechnol.* **7,** 394 (2012).

17 Wu, G., More, K. L., Johnston, C. M. & Zelenay, P. High-performance electrocatalysts for oxygen reduction derived from polyaniline, iron, and cobalt. *Science* **332,** 443-447 (2011).

18 Li, Q. *et al.* Graphene/graphene-tube nanocomposites templated from cage-containing metal-organic frameworks for oxygen reduction in Li–O_2 batteries. *Adv. Mater.* **26,** 1378-1386 (2014).

19 Yang, G., Choi, W., Pu, X. & Yu, C. Scalable synthesis of bi-functional high-performance carbon nanotube sponge catalysts and electrodes with optimum C–N–Fe coordination for oxygen reduction reaction. *Energy*

Environ. Sci. **8,** 1799-1807 (2015).

20 Fu, X. *et al.* Co–N decorated hierarchically porous graphene aerogel for efficient oxygen reduction reaction in acid. *ACS Appl. Mater. Interfaces* **8,** 6488-6495 (2016).

21 Ferrero, G. A. *et al.* Fe–N-doped carbon capsules with outstanding electrochemical performance and stability for the oxygen reduction reaction in both acid and alkaline conditions. *ACS Nano* **10,** 5922-5932 (2016).

22 Han, Y. *et al.* Hollow N-doped carbon spheres with isolated cobalt single atomic sites: superior electrocatalysts for oxygen reduction. *J. Am. Chem. Soc.* **139,** 17269-17272 (2017).

23 Chung, H. T. *et al.* Direct atomic-level insight into the active sites of a high-performance PGM-free ORR catalyst. *Science* **357,** 479-484 (2017).

24 Yang, L. *et al.* Unveiling the high-activity origin of single-atom iron catalysts for oxygen reduction reaction. *PNAS.* **115,** 6626-6631 (2018).

25 Pan, Y. *et al.* A bimetallic Zn/Fe polyphthalocyanine-derived single-atom Fe-N_4 catalytic site:a superior trifunctional catalyst for overall water splitting and zn–air batteries. *Angew. Chem. Int. Ed.* **57,** 8614-8618 (2018).

26 Jiao, L. *et al.* From metal–organic frameworks to single-atom Fe implanted N-doped porous carbons: efficient oxygen reduction in both alkaline and acidic media. *Angew. Chem. Int. Ed.* **57,** 8525-8529 (2018).

27 Han, A. *et al.* A polymer encapsulation strategy to synthesize porous nitrogen-doped carbon-nanosphere-supported metal isolated-single-atomic-site catalysts. *Adv. Mater.* **30,** 1706508 (2018).

28 Wu, H. *et al.* Highly doped and exposed Cu(I)–N active sites within graphene towards efficient oxygen reduction for zinc–air batteries. *Energy Environ. Sci.* **9,** 3736-3745 (2016).

29 Fu, X. *et al.* In situ polymer graphenization ingrained with nanoporosity in a nitrogenous electrocatalyst boosting the performance of polymer-electrolyte-membrane fuel cells. *Adv. Mater.* **29,** 1604456 (2017).

30 Zamani, P. *et al.* Highly active and porous graphene encapsulating carbon nanotubes as a non-precious oxygen reduction electrocatalyst for hydrogen-air fuel cells. *Nano Energy* **26,** 267-275 (2016).

31 Stariha, S. *et al.* PGM-free Fe-N-C catalysts for oxygen reduction reaction: catalyst layer design. *J. Power Sources* **326,** 43-49 (2016).

32 Peng, H. *et al.* Ultra-high-performance doped carbon catalyst derived from o-phenylenediamine and the probable roles of Fe and melamine. *Appl. Catal., B* **158-159,** 60-69 (2014).

33 Nabae, Y. *et al.* High performance Pt-free cathode catalysts for polymer electrolyte membrane fuel cells prepared from widely available chemicals. *J. Mater. Chem. A* **2,** 11561-11564 (2014).

34 Tian, J. *et al.* Optimized synthesis of Fe/N/C cathode catalysts for pem fuel cells: a matter of iron–ligand coordination strength. *Angew. Chem. Int. Ed.* **125,** 7005-7008 (2013).

35 Bashyam, R. & Zelenay, P. A class of non-precious metal composite catalysts for fuel cells. *Nature* **443,** 63 (2006).
